# Hydroflux Synthesis and Structural Phase Transition of Rare‐Earth Borate Hydroxides Na_2_[*RE*(BO_3_)(OH)_2_] (*RE*=Y, Gd−Er)

**DOI:** 10.1002/chem.202402783

**Published:** 2024-10-16

**Authors:** Yuxi Li, Eduardo Carrillo‐Aravena, Jiang Qu, Gohil S. Thakur, Michael Ruck

**Affiliations:** ^1^ Faculty of Chemistry and Food Chemistry Technische Universität Dresden 01062 Dresden Germany; ^2^ Würzburg-Dresden Cluster of Excellence ct.qmat Technische Universität Dresden 01062 Dresden Germany; ^3^ Leibniz Institute for Solid State and Materials Research Dresden Helmholtzstraße 20 01069 Dresden Germany; ^4^ Department of Chemical Sciences Indian Institute of Science Education and Research Berhampur Berhampur 760010 India; ^5^ Max Planck Institute for Chemical Physics of Solids Nöthnitzer Straße 40 01187 Dresden Germany

**Keywords:** Crystal structure, Hydroflux synthesis, Hydroxometalates, Boron compounds, Rare earth compounds

## Abstract

Reacting *RE*_2_O_3_ and H_3_BO_3_ in an ultra‐alkaline NaOH hydroflux at about 250 °C yielded pure, crystalline samples of Na_2_[*RE*(BO_3_)(OH)_2_] (*RE*=Y, Gd−Er). The compounds dehydrate to Na_3_ *RE*(BO_3_)_2_ upon heating in air to about 500 °C. Na_2_[*RE*(BO_3_)(OH)_2_] (*RE*=Tb−Er) are photoluminescent under UV radiation. Their UV‐Vis spectra show the typical absorptions associated with 4*f*–4*f* transitions and absorption edges in the UV (band gaps ≥5.7 eV). The *RE* ^3+^ cation is coordinated by seven oxygen atoms, which define a distorted pentagonal bipyramid. The bipyramids share *trans*‐edges of their base forming infinite chains. Triangular (BO_3_)^3−^ groups connect the chains into layers. The crystal structure of Na_2_[Ho(BO_3_)(OH)_2_] was investigated at various temperatures in the range 100 K≤*T*≤320 K. Above 310(2) K, the compound crystallizes in the orthorhombic space group *Cmcm* (β‐phase), below, it undergoes a displacive phase transition of second order resulting in a monoclinic structure in space group *C*2/*c* (α‐phase). The critical exponents derived from different structural parameters indicate a cooperative distortion of the borate layers, but a rather uncorrelated adaptation of the sodium cations to their local environment. The other compounds of the series also adopt the structure of the α‐phase at 296 K.

## Graphical abstract

A new family of rare‐earth borates is crystallized from an ultra‐alkaline NaOH hydroflux at moderate temperature. Pentagonal bipyramids centered by RE^3+^ cations form chains that are connected into layers by triangular (BO_3_)^3−^ groups. Slightly above room temperature, the structure undergoes a second‐order phase transition. Some of the compounds are photoluminescent under UV radiation.

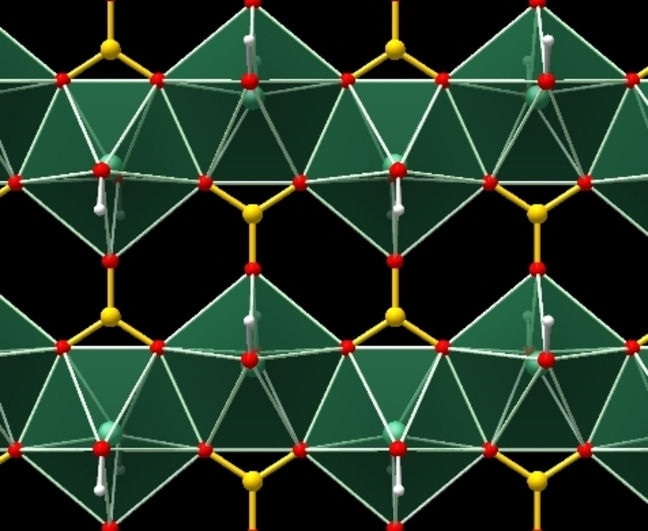

## Introduction

Borate anions of various types occur in many minerals, and because of their great structural diversity and useful properties, such as negative thermal expansion or nonlinear optical behavior, many more synthetic borates have been investigated.^[1]^ The boron atoms are bonded to two, three or four oxygen atoms, resulting in the linear (BO_2_)^−^ anion,^[2]^ planar (BO_3_)^3−^ triangles, or (BO_4_)^5−^ tetrahedra. Edge‐ and corner‐sharing of these groups create more complex anions up to frameworks. All types of metal cations as well as protons can be used to balance the charge, albeit the bonding forms between the metal and oxygen atoms of the borate anions can differ significantly. Of particular interest are borates of the rare‐earth metals (*RE*), as many of these elements can add further optical properties due to their 4*f*–4*f* electronic transitions.[^3^, ^4^, ^5^, ^6^, ^7^, ^8^] Examples of salts that are based on the (BO_3_)^3−^ are Li_3_ *RE*(BO_3_)_2_,[^9^, ^10^] Na_3_Y_3_(BO_3_)_4_,^[11]^ Na_3_ *RE*(BO_3_),^[12]^ NaNd[B_6_O_9_(OH)_4_],^[13]^ Na_2_Ce_2_[BO_2_(OH)(BO_3_)]_2_ ⋅ H_2_O,^[14]^ K_3_Li_3_ *RE*_7_(BO_3_)_9_,^[15]^ and K_3_ *RE*_3_(BO_3_)_4_.^[16]^ One motivation for our study was that sodium‐containing borates are among the most popular fluorescent agents.[^17^, ^18^, ^19^] The recent discovery of alkaline and/or alkaline earth rare‐earth borate compounds has prompted more research to achieve high luminescence efficiency.[^16^, ^20^, ^21^]

There are several approaches for synthesizing borates, including high‐temperature solid‐state reactions, hydrothermal reactions, and flux crystal growth. In many cases, a long reaction time combined with high temperatures and in some cases high pressure are needed to grow good quality crystals. For example, crystals of the nonlinear optical material Na_3_La_2_(BO_3_)_3_ have been obtained with the top‐seeded solution growth method,^[22]^ and Na_2_Ce_2_[BO_2_(OH)][BO_3_]_2_ ⋅ H_2_O crystals have been grown by hydrothermal reactions, which duration of the experiments was 18–20 days.^[14]^ Li_3_La_2_(BO_3_)_3_ crystals have been grown at 800 °C in a sealed silica tube.^[23]^

We are currently exploring the potential of ultra‐alkaline aqueous media, known as hydroflux,^[24]^ for the facile and resource‐efficient synthesis of functional inorganic materials and, in particular, are testing the applicability of the method for the preparation of rare‐earth borates. In a hydroflux synthesis, water is mixed with hydroxides of alkali or alkaline earth metals in typical molar ratios of 1 : 1–2 : 1, resulting in approximately 50–25 molar (m) solutions. The hydroflux method differs from hydrothermal conditions due to the extremely low water activity, which enables almost pressure‐free syntheses at approximately 200 °C and also the formation of moisture‐sensitive products. The reaction takes only a few hours and is best carried out in an autoclave lined with PTFE (polytetrafluoroethylene), which is inert to the alkaline medium and guarantees that no water is lost during the reaction. The alkaline hydroflux readily dissolves most oxides,[^25^, ^26^, ^27^] and has proven useful in the syntheses of diverse crystalline metal oxides and hydroxides,[^27^, ^28^, ^29^, ^30^, ^31^, ^32^, ^33^, ^34^, ^35^, ^36^] including application to battery electrode materials.^[37]^

The first application of the method to borates has yielded the new compound Ba(BO_2_(OH)).^[38]^ Here we report the synthesis, crystal structures, and selected properties of the rare‐earth borate hydroxides Na_2_[*RE*(BO_3_)(OH)_2_] with *RE*=Y, Gd−Er.

### Synthesis

**Figure 1:**
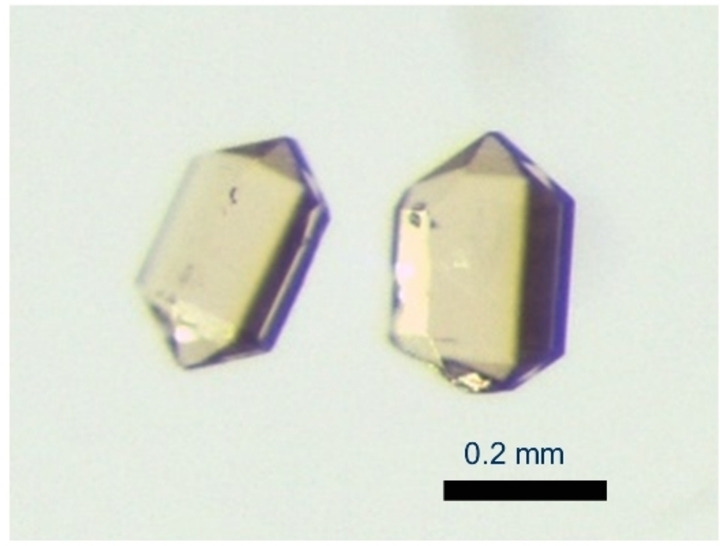
Representative Na_2_[Ho(BO_3_)(OH)_2_] crystals grown from alkaline hydroflux.

To synthesize Na_2_[*RE*(BO_3_)(OH)_2_] (*RE*=Y, Gd−Er) boric acid B(OH)_3_ and rare‐earth oxides *RE*_2_O_3_ were carefully added to the aqueous sodium hydroxide solution that constitutes the hydroflux. Transparent crystals of Na_2_[*RE*(BO_3_)(OH)_2_] (Figures 1 and S1) were obtained after reaction of the mixture at 250 °C for 10 h in a stainless steel autoclave with PTFE inlet (Equation 1).
(1)

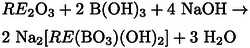




The Na_2_[*RE*(BO_3_)(OH)_2_] compounds dissolve in diluted alkaline solution or water, however they withstand quick washing with methanol (Figure S2). The compounds are not strongly hygroscopic or air‐sensitive (Figures S1 and S3), but the surface becomes rough after two months of storage in air (Figure S2). The final yields were 94 % (Y), 83 % (Gd), 91 % (Tb), 89 % (Dy), 97 % (Ho), and 94 % (Er).

In agreement with the literature,^[39]^ the base concentration of the hydroflux was found to be an important reaction parameter. For example, high‐quality crystals of Na_2_[Ho(BO_3_)(OH)_2_] were obtained when using a 40 m hydroflux (Figures 1 and S4). By lowering the concentration to 20 m, the size and the quality of the crystals decreased and Ho(OH)_3_ formed as a by‐product. At 50 m, Ho_2_O_3_ is found after the reaction. The best single‐crystals in terms of size and quality for diffraction experiments were obtained with water to base ratios *q*(Na)=*n*(H_2_O): *n*(NaOH) of 1.4 (Gd, Tb, Dy, Ho), 1.85 (Er), and 2.78 (Y).

**Figure 2:**
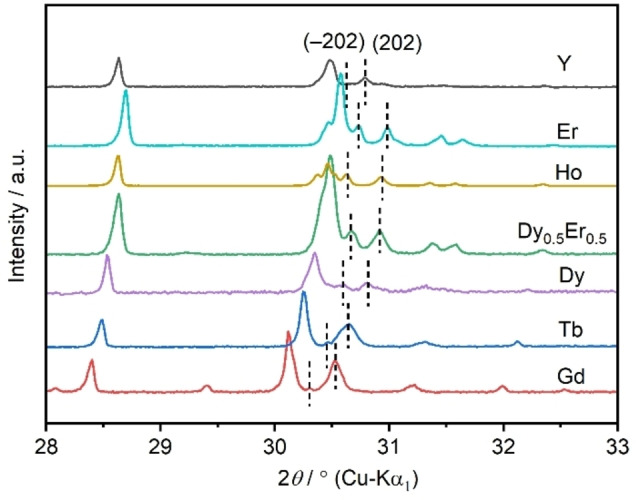
Sections of the PXRD patterns of Na_2_[*RE*(BO_3_)(OH)_2_] (*RE*=Y, Gd−Er) at 296 K. The different diffraction angels of the reflections (−202) and (202) indicate that the angle *β* deviates from 90°.

The powder X‐ray diffraction (PXRD) patterns of washed Na_2_[*RE*(BO_3_)(OH)_2_] samples match the calculated patterns and show no additional reflections (Figures 2 and S3). The lattice parameters show a linear dependence on the ionic radii of the seven‐coordinated *RE* ^*3+*^ cations (Figure S5). We did not obtain Na_2_[*RE*(BO_3_)(OH)_2_] compound of the other rare‐earth elements, which suggests that this structure type is restricted to seven‐coordinated rare earth ions with tabulated radii between 100 and 94.5 pm.^[40]^

To test whether solid solutions can form, the mixed phase Na_2_[Dy_0.5_Er_0.5_(BO_3_)(OH)_2_] was targeted using equimolar amounts of Er_2_O_3_ and Dy_2_O_3_ and *q*(Na)=1.4. Single‐phase Na_2_[Dy_0.5_Er_0.5_(BO_3_)(OH)_2_] with a yield of 87 % was obtained. Semi‐quantitative energy‐dispersive X‐ray (EDX) analysis (Table S1) showed a ratio Er: Dy=0.84(4): 1.00(5) and a homogeneous distribution of both elements in the crystal (Figure S6). The powder X‐ray diffraction (PXRD) pattern showed no broadening or splitting of reflections (Figures S3 and S7), indicating isomorphism.

### Crystal Structures

**Figure 3:**
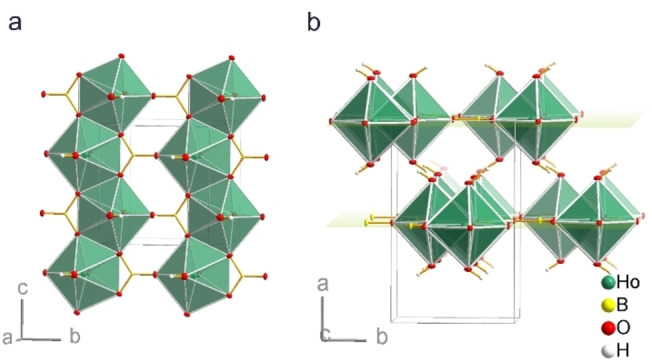
(a) Section of the crystal structure of β‐Na_2_[Ho(BO_3_)(OH)_2_] with infinite chains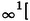 HoO_1/1_O_4/2_(OH)_2/1_] of edge‐sharing pentagonal bipyramids that run along the [001] direction. Planar (BO_3_)^3−^ anions connect the chains into layers. (b) Stacking of 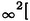 Ho(BO_3_)(OH)_2_]^2−^ layers along the [100] direction.

Single‐crystal diffraction data analysis reveals that all compounds Na_2_[*RE*(BO_3_)(OH)_2_] (*RE*=Y, Gd−Er) are isostructural and show at least two temperature‐dependent polymorphs. The high temperature structure (β‐phase) was determined at 310(2) K and crystallizes in the orthorhombic space group *Cmcm* (no. 63; Tables 1, S3, S6 and S15). The polymorphs stable at room temperature (296 K) and below (α‐phase; Figure 3, S8) were measured at 100 K. They crystallize in the monoclinic space group *C*2/*c* (no. 15; Table 1, S2 and S7 to S14). All investigated monoclinic crystals were twinned along [100], some also along [001].

**Table 1:** Lattice parameters of α‐Na_2_ *RE*[(BO_3_)(OH)_2_] (*RE*=Y, Gd−Er) at 100 K and β‐Na_2_Ho[(BO_3_)(OH)_2_] at 310 K (in italics).

*RE*	*a*/pm	*b*/pm	*c*/pm	*β*/°	*V*/(10^6^ pm^3^)
Gd	1066.83(4)	655.43(3)	694.41(3)	92.263(3)	485.18(4)
Tb	1061.70(1)	651.23(1)	690.52(3)	92.258(3)	477.03(1)
Dy	1061.55(3)	649.97(2)	689.59(2)	92.235(2)	475.44(2)
Ho	1056.85(2) *1064.61(9)*	646.40(1) *645.74(8)*	686.64(1) *690.76(8)*	92.174(2) *90*	468.74(1) *474.88(9)*
Er	1055.23(2)	643.81(1)	685.31(1)	92.093(1)	465.27(1)
Dy,Er	1057.50(3)	646.03(2)	687.02(2)	92.101(3)	469.04(2)
Y	1058.31(6)	645.98(3)	689.43(4)	92.048(5)	471.03(4)

**Figure 4:**
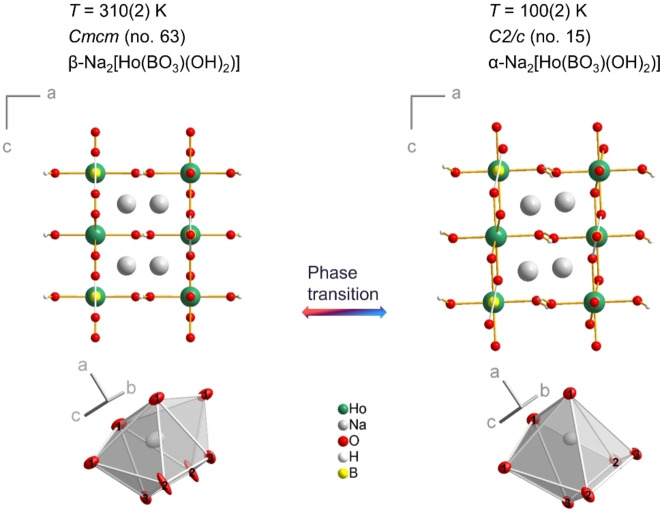
Structure of Na_2_[Ho(BO_3_)(OH)_2_] and coordination polyhedron of the Na^+^ cation in the orthorhombic β‐phase at 310 K (left) and in the monoclinic α‐phase at 100 K (right). The ellipsoids enclose a space in which with 95 % probability the electron density of the atoms can be found.

We discuss the crystal structures using the holmium compound as an example (Figure 3 and 4). In the orthorhombic β‐polymorph, the holmium(III) atom centers a distorted pentagonal bipyramid formed by seven oxygen atoms (Figure S9). The basal oxygen atoms belong to borate groups (BO_3_)^3−^, the apical oxygen atoms are protonated (hydroxide groups). The Ho−O bond lengths range from 227.1(2) to 236.6(2) pm (average 231.8 pm). The bipyramids share two *trans*‐edges of their basal five‐membered rings forming undulated rods 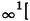 HoO_1/1_O_4/2_(OH)_2/1_], which run parallel to the *c* axis. The shortest Ho⋅⋅⋅Ho distance is 383.4(1) pm. A similar coordination motif was found, for example, in BiSr_3_(YO)_3_(BO_3_)_4_.^[41]^ The planar (BO_3_)^3−^ anion lies on the mirror plane perpendicular to the [100] direction. It connects two adjacent rods by acting as an end‐on monohapto ligand on one side and as a dihapto ligand bridging two cations of the same rod on the other side. As a result, layers [Ho(BO_3_)(OH)_2_]^2−^ parallel to the (100) plane are formed. Weak hydrogen bonds O1−H⋅⋅⋅O1 (155°, O1⋅⋅⋅O1 333 pm) connect the layers. The axis ratio *a*/*b*=1.645 indicates that the three‐dimensional arrangement of the rods does not differ much from a hexagonal packing (*a*/*b*=1.732). The sodium cations occupy voids between the layers with Na−O distances between 240.2(2) and 284.1(2) pm (average 262.1 pm; Figure 3).

A continuous second‐order phase transition occurs upon decreasing the temperature (Figures S8 and S10, Tables S5, S14 and S15). Below 310 K, the angle *β* deviates of from 90°. The reduction in symmetry corresponds to a transition of index 2 from the orthorhombic space group *C* 2/*m* 2/*c* 2_1_/*m* to its maximum *translationengleiche* monoclinic subgroup *C* 1 2/*c* 1 (see Figure S11 for the Bärnighausen tree). Twinning, which indirectly preserves the lost symmetry, is almost unavoidable for structural phase transitions of this type and also occurs here.

In the crystal structure, the (BO_3_)^3−^ anion tilts around the B−O3 bond, i. e. [010], away from the (100) plane, breaking mirror symmetry. However, it remains planar with a sum of O−B−O angles of 360.0(7)°. Although this creates a distortion of the basal five‐membered ring of the holmium‐centered bipyramid, the Ho−O distances do not change significantly. At 100 K, the Ho−O bond lengths are slightly reduced (−0.4 pm) compared with the β‐phase structure at 310 K and range from 226.7(2) to 236.8(2) pm. The shortest Ho⋅⋅⋅Ho distance is 381.9(1) pm. The sodium cations, however, show a clear deviation from the twofold rotation axes parallel to [100]. This shift moves the sodium cation from the center of its coordination polyhedron and decreases its coordination number from eight to seven. The Na−O distances at 100 K range from 237.8(3) to 276.1(2) pm, with an average that is considerably shorter (−6.9 pm) than at room temperature.

The thermal expansion of Na_2_[Ho(BO_3_)(OH)_2_], including the phase transition, is Δ*a*/(*a ⋅* Δ*T*)=3.9 ⋅ 10^−5^ K^−1^, Δ*b*/(*b ⋅* Δ*T*)=4.9 ⋅ 10^−6^ K^−1^, and Δ*c*/(*c ⋅* Δ*T*)=2.8 ⋅ 10^−5^ K^−1^. The smallest change is seen in the *b* direction, where the layer is rigid. The inclination of the (BO_3_)^3−^ anion shortens the *c* axis, but an even stronger effect on the packing is found along the stacking direction of the layers, i. e. the *a* axis. This also leads to increased hydrogen bonding, including not only O1−H⋅⋅⋅O1 (142°, O1⋅⋅⋅O1 333 pm) but also O1−H⋅⋅⋅O2 (137°, O1⋅⋅⋅O2 320 pm). Overall, the main driving force for the structural phase transition appears to be the temperature‐dependent space requirement of the sodium cation.

A series of data sets at different temperatures allowed categorizing the phase transition as second‐order. According to the Landau theory, the order parameter *η* follows a power law below the critical temperature *T*_c_ and becomes zero at *T*_c_ (Equation 2).

(2) $$\eta =A{\left(\frac{T_{c}-T}{T_{c}}\right)}^{b}$$

*A* is a constant, and *b* is the critical exponent. We considered different order parameters: the deviation of the angle *β* from 90° (*η*=*β*–90°) as well as the shifts of the atoms O1 and O2 (*η*=[(*x*–*x*_c_)^2^+(*y–y*_c_)^2^+(*z–z*_c_)^2^]^1/2^). The evaluation of the order parameter curves revealed *T*_c_=306(1) °C and *b* between 0.41 and 0.47 (Figure S12). A critical exponent close to 1/2 suggests long‐range interactions as the driving force for the structural distortion, or in this context, a cooperative distortion of the borate layers. In contrast, the critical exponent derived from the shift of the Na atom is 0.34, i. e. close to 1/3, which generally stands for short‐range interactions. Apparently, the adaptation of the isolated sodium cations to their local environment does not occur in a correlated manner. This can be understood by their high coordination number and the predominantly non‐covalent interactions with their environment.

Repeated measurements of a Na_2_[Ho(BO_3_)(OH)_2_] sample with a differential scanning calorimeter (DSC) showed reproducibly a small thermal effect between 300 and 310 K, which evidences a change of the specific heat capacity during the phase transition (Figure S13). For the other compounds, it can be deduced from their monoclinic angles at 296 K, which show only slight deviations from 90° (Table S4), that their phase transition temperatures should be quite similar to that of the Ho compound.

### Thermal Decomposition

**Figure 5:**
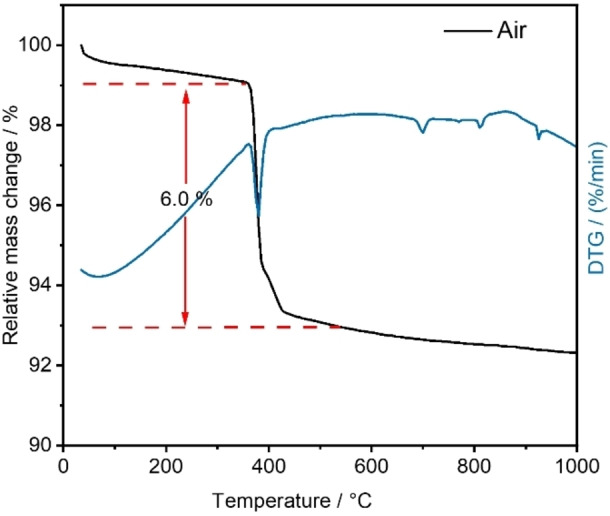
Thermogravimetric analysis of Na_2_[Dy(BO_3_)(OH)_2_] in synthetic air at a heating rate of 5 K min^−1^.

Combined differential thermal analyses (DTA) and thermogravimetric (TG) analyses were performed to study the thermal stability of these compounds. As a representative example, the thermogravimetry curve for Na_2_[Dy(BO_3_)(OH)_2_] heated in a stream of CO_2_‐free synthetic air is shown in Figure 5. A continuous weight loss of about 1 % of the initial mass was observed until the first significant effect occurred at about 360 °C. The loss of 6 wt.% corresponds to the evaporation of one water molecule per formula unit (calculated 5.98 wt.%). No further significant weight loss was observed up to a temperature of 1000 °C. The TG curve for an experiment using an argon stream was similar (Figure S14), ruling out any influence of oxygen on decomposition. To determine the product of the thermal treatment, a Na_2_[Dy(BO_3_)(OH)_2_] sample was annealed in a crucible open to the atmosphere at 500 °C for 24 h. The PXRD pattern revealed Na_3_Dy(BO_3_)_2_ and Dy_2_O_3_ (Figure S15) as crystalline products of the dehydration process (Equation 3).
(3)

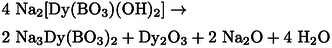




The solid product exhibited cracks on its surface, but the initial crystal morphology was well preserved (Figure S16). To test for Na_2_O, or more specifically for NaOH formed with atmospheric moisture, Na_2_Dy[(BO_3_)(OH)_2_] and the decomposition product were simultaneously placed on pH test papers and left in air for 2 h. Only the annealed sample showed an alkaline reaction (Figure S17).

### Spectroscopic Characterization

**Figure 6:**
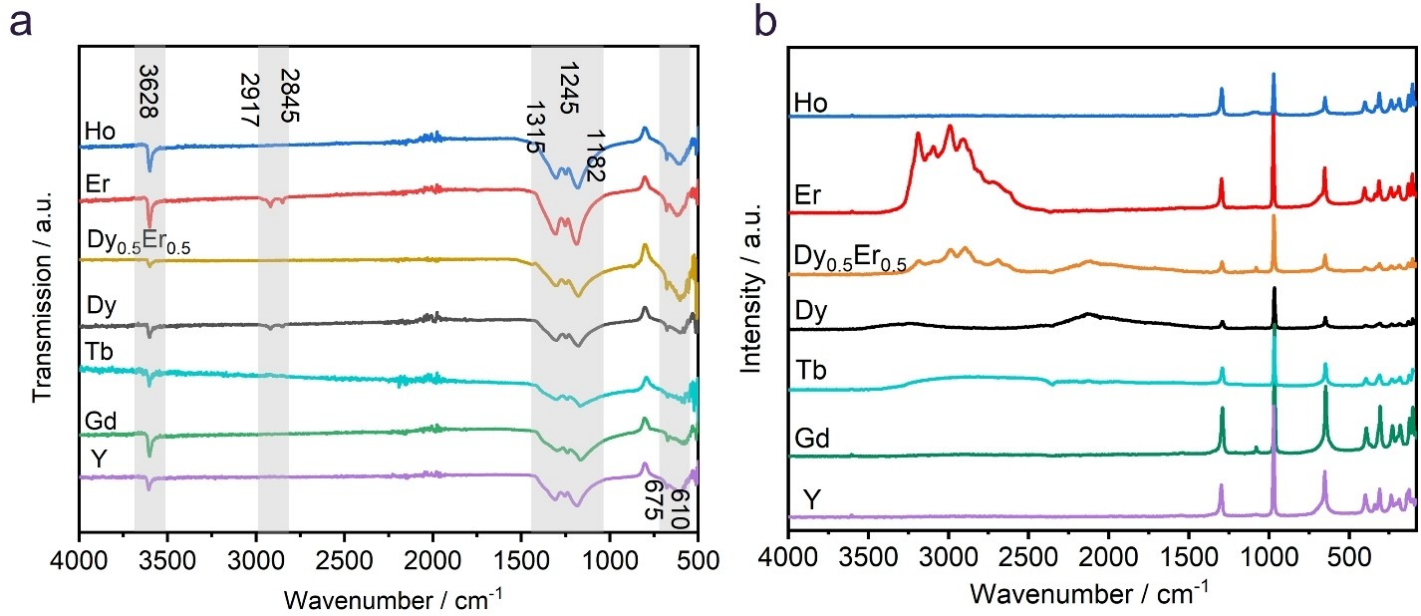
(a) ATR‐FT‐IR and (b) Raman spectra of Na_2_[*RE*(BO_3_)(OH)_2_] (*RE*=Y, Gd−Er) measured with a 1064 nm laser at room temperature. The weak peak around 1070 cm^−1^ is probably due to traces of Na_2_CO_3_ on the surface.

The infrared (IR) spectra at room temperature of all Na_2_[*RE*(BO_3_)(OH)_2_] compounds (Figure 6) show absorption bands around 3628 cm^−1^ which can be attributed to the O−H stretching mode.^[42]^ In some of the spectra, weak absorption is found also at 2917 and 2845 cm^−1^, which is typical for NaOH, probably a residue from the hydroflux. The vibrational modes of the (BO_3_)^3−^ anion are found between 1500 and 1100 cm^−1^ (asymmetric stretching) and between 675 and 610 cm^−1^ (bending) in accordance with the literature.[^43^, ^44^, ^45^, ^46^, ^47^, ^48^, ^49^] Bands at lower frequencies are assigned to lattice dynamics, including Na−O and *RE*−O stretching modes.

Additional undetermined bands appear in the FTIR and particularly in the Raman spectra due to fluorescence arising from 4*f*–4*f* transitions of the *RE* ^3+^ cations. Therefore, we used excitation lasers with different wavelengths for the Raman spectroscopy (Figures 6 and S18), to separate fluorescence from the vibrational absorption bands. The Raman spectra show sharp signals that can be assigned to the borate anion (asymmetric stretching at 1300 cm^−1^, symmetric stretching at 980 cm^−1^, bending at 600–700 cm^−1^). The vibrational characteristics of the borate anion group observed in the Raman spectra are in good agreement with the FTIR spectra.

**Figure 7:**
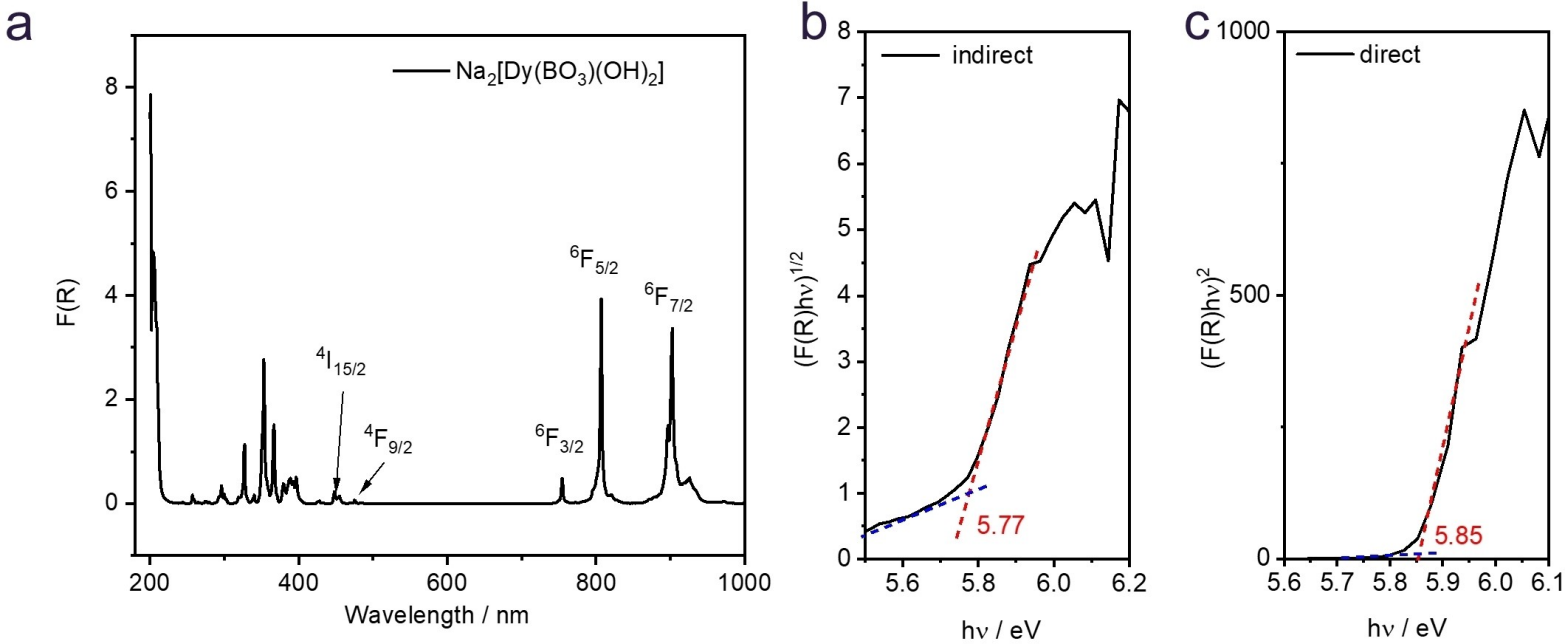
UV‐Vis diffuse reflectance spectrum of Na_2_[Dy(BO_3_)(OH)_2_] (a) and baseline corrected Tauc plots for indirect (b) and direct (c) optical transitions.

The transparent, only weakly colored appearance of the crystals suggest that the band gaps of all Na_2_[*RE*(BO_3_)(OH)_2_] compounds lie in the UV region. The measured absorption edges in the UV‐Vis diffuse reflectance spectra (Figures 7 and S19–S23) were transformed using the Kubelka‐Munk function and analyzed with Tauc plots [F(*R*_∞_)h*v*]^1/n^.^[50]^ The estimated band gaps assuming indirect allowed transitions (*n*=2) were 5.91, 5.85, 6.05, 5.77, 5.91, 5.86 eV for *RE*=Y, Gd, Tb, Dy, Ho, Er and 6.00, 6.01, 5.85, 5.85, 6.03, 6.01 eV assuming direct allowed transitions (*n*=0.5). Despite the limited applicability of this method,^[51]^ it can be stated that the rare‐earth borates presented here have very wide band gaps (similar to diamond) and are UV transmissive down to about 220 nm. This makes especially the colorless yttrium compound an interesting material for UV‐C windows.

Apart from the band gap absorption edge in the UV, the UV/Vis spectra of Na_2_[*RE*(BO_3_)(OH)_2_] are governed by distinctive electronic transitions (Figures 7 and S19–S23). According to the energy diagrams proposed by *Dieke* and *Carnall*,[^52^, ^53^] the *f*‐*f* and *f*‐*d* transitions of the *RE* ^*3+*^ cations exhibit characteristic absorption peaks between 200 and 1000 nm. Na_2_[Dy(BO_3_)(OH)_2_] exhibits strong absorption bands at 454, 475, 755, 807, and 901 nm (Figure 7), which usually correspond to *f*‐*f* transitions from the ground state ^6^H_15/2_ to the excited states ^4^I_15/2_, ^4^F_9/2_, ^6^F_3/2_, ^6^F_5/2_ and ^6^F_7/2_. Although Na_2_[Dy(BO_3_)(OH)_2_] shows absorptions in the red and blue to violet range, the compound does not appear to have a color visible to the naked eye. Na_2_[Er(BO_3_)(OH)_2_] has a pink color in daylight, associated with absorption bands in the visible range at 409, 452, 489, 522, 546, 653 and 801 nm (from ^4^I_15/2_ to ^2^H_9/2_, ^4^F_5/2_, ^4^F_7/2_, ^2^H_11/2_, ^4^S_3/2_, ^4^F_9/2_ and ^4^I_9/2_). The light yellow Na_2_[Ho(BO_3_)(OH)_2_] absorbs at 420, 455, 475, 539, and 641 nm (from ^5^I_8_ to ^5^G_5_, ^5^G_6_+^5^F_1_, ^5^F_3_, ^5^F_4_+^5^S_2_, and ^5^F_5_).^[54]^ Na_2_[Tb(BO_3_)(OH)_2_] exhibits weak absorption bands at 486, 378, 366 and 358 nm have been assigned to the transitions from the ground state ^7^F_6_ to the higher excited levels of ^5^D_4_, ^5^D_3_+^5^G_6_, ^5^L_10_, and ^5^L_9_+^5^G_4_.^[55]^ The compounds with *RE*=Y or Gd do absorb significantly in the visible range, which renders them colorless.

**Figure 8:**
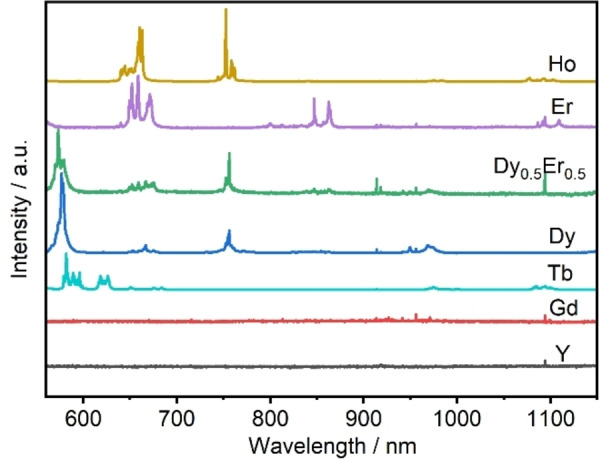
Photoluminescence emission spectra comparison of Na_2_[*RE*(BO_3_)(OH)_2_] (*RE*=Y, Gd−Er) on Si under 458 nm excitation.

The photoluminescence spectra of the crystals under the excitation laser photon wavelength (458 nm) are shown in Figure 8. The sample shows characteristic emission peaks due to Stark splitting of the degenerate 4 *f* levels. The spectrum of Na_2_[Ho(BO_3_)(OH)_2_] exhibits characteristic emissions attributed to the transitions in Ho^3+^ ions: the peak around 650 nm due to the ^5^F_5_→^5^I_8_ transition and the near infrared (NIR) luminescence band, situated around 754 nm due to the (^5^F_2_, ^5^F_4_) ^5^I_7_ transition.^[56]^ There are two main ranges wave bands of Er^3+^ are ^4^F_9/2_→^4^I_15/2_ at around 660 nm^[57]^ and ^4^S_3/2_→^4^I_13/2_ at around 850 nm. The sample with Dy^3+^ has the characteristic emission bands at around 587 nm and 750 nm related to the ^4^F_9/2_→^6^H_13/2_, ^4^F_9/2_→(^6^H_9/2_, ^6^F_11/2_) transition.^[58]^ The peak at around 586 nm and 620 nm of Tb^3+^ are associated with the ^5^D_4_→^7^F_4_ and ^5^D_4_^7^F_3_.^[59]^ The photoluminescence spectra of the elements of Gd and Y have weak signals around 1090 nm in the NIR range. Crystals of Na_2_[Y(BO_3_)(OH)_2_] doped with 1 % Ho^3+^, showed no fluorescence under UV light.

### Magnetism

**Figure 9:**
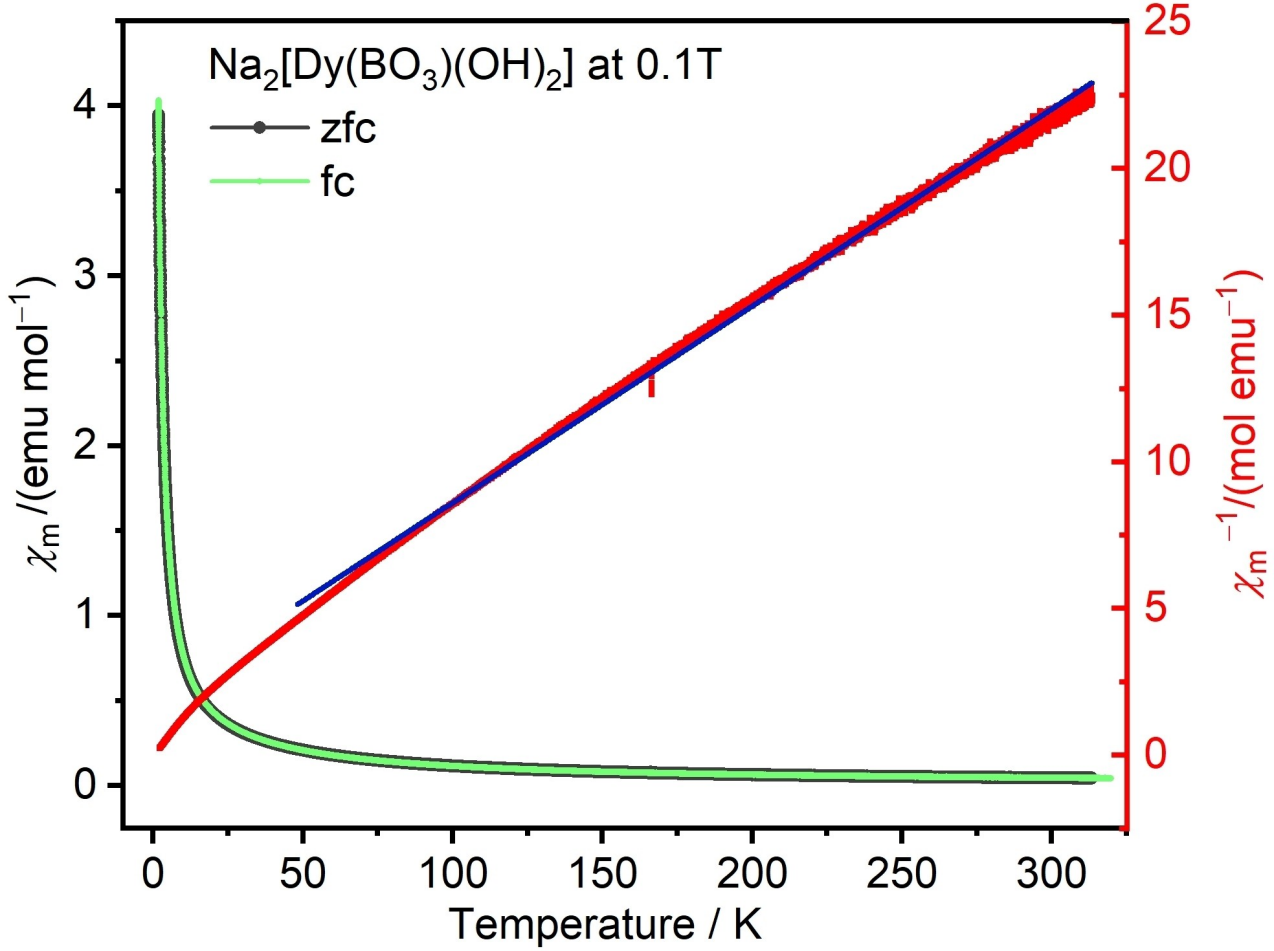
Molar magnetic susceptibility of Na_2_[Dy(BO_3_)(OH)_2_] and its inverse measured in a field of μ_0_ *H*=0.1 T between 2 and 300 K. The blue line is a linear Curie‐Weiss fit in the temperature region between 50 and 325 K.

Some of the here employed *RE* ^3+^ cations have very high magnetic moments. The magnetic susceptibility of Na_2_[Dy(BO_3_)(OH)_2_] in the temperature range between 2 K and 300 K and in an external field of μ_0_ *H*=0.1 T in both zero‐field‐cooled (zfc) and field‐cooled (fc) protocols (warming runs) is shown in Figure 9. There is no bifurcation in the zfc and fc datasets, and no anomalies are observed in the entire temperature range indicating that there is no magnetic phase transition or short range magnetic correlation, thus the compound is essentially paramagnetic. Paramagnets are expected to follow the Curie‐Weiss law χ=*C*/(*T*–*θ*_w_), where *C* is the Curie constant relating to the magnitude of the magnetic moments and *θ*_W_ is the Weiss constant that can provide information about the type and strength of magnetic interactions. The inverse susceptibility data in the temperature range between 50 and 325 K fairly fit to a straight line, yielding *C*=15.0 emu mol^−1^ K^−1^ and *θ*_W_=−30 K. The latter suggests weak antiferromagnetic exchange interactions between the rare‐earth cations. However, the do not result in cooperative magnetism above 2 K. This is consistent with the large distance of 383.4(1) pm between the magnetic ions in the crystal structure. The experimental effective paramagnetic moment for Na_2_[Dy(BO_3_)(OH)_2_] is calculated to be *P*_eff_=10.9 *μ*_B_ per formula unit, which is very close to the expected value of 10.7 *μ*_B_ for the Dy^3+^ cation.^[60]^ Several references have shown that the cations in mixed rare‐earth compounds can influence each other's effective magnetic moments.[^61^, ^62^] Nevertheless, the effective magnetic moments of Na_2_[Dy_0.5_Er_0.5_(BO_3_)(OH)_2_] of 10.5 *μ*_B_ per formula unit (Figure S24) is also very close to the expected mean of 10.2 *μ*_B_ for an equimolar mixture of Dy^3+^ (10.7 *μ*_B_) and Er^3+^ (9.6 *μ*_B_).

## Conclusions

Using alkaline hydroflux as reaction medium we succeeded in synthesizing a new family of rare earth borates. The compounds Na_2_[*RE*(BO_3_)(OH)_2_] (*RE*=Y, Gd−Er) crystallize isostructural and show a transition from an orthorhombic high‐temperature phase to a monoclinic lattice just above room temperature. The displacive phase transition is of second‐order and interestingly combines cooperative distortion and uncorrelated ordering of different structural components. The crystals proved to be stable in dry air up to 350 °C. They have wide band gaps of up to 6 eV and are UV transmissive down to about 220 nm, which could be interesting for application in UV technology. The rare‐earth cations with *f*‐*f* transitions show the typical absorption and luminescence properties. The compounds including magnetic cations are paramagnetic down to 2 K.

## Experimental Section

### Synthesis

Na_2_[*RE*(BO_3_)(OH)_2_] was synthesized in a NaOH hydroflux. The reaction was carried out in a PTFE‐lined 50 mL Berghof type DAB‐2 autoclave to prevent water loss. In the optimized synthesis protocol, different compound has different molar water‐base ratio of 1.4 : 1 (*RE*=Gd−Ho), 1.85 : 1 (Er) and 2.78 : 1 (Y). Starting from 3 mmol H_3_(BO_3_) (99.99 %, abcr), 0.5 mmol *RE*_2_O_3_ [*RE*=Y (Fluka, 99.98 %), Gd (99.9 %, Alfa), Tb (99.99 %, sigma) Dy (99.9 %, Riedel‐de Haën), Ho (99.995 %, abcr), Er (99.9 %, abcr)] and 3 mL deionized water, NaOH (99 %, Grüssing GmbH) was added in the amount of 4.8 g (Gd−Ho), 3.6 g (Er), or 2.4 g (Y). For the syntheses of Na_2_[Dy_0.5_Er_0.5_(BO_3_)(OH)_2_], 0.25 mmol Er_2_O_3_ and 0.25 mmol Dy_2_O_3_ were combined with 3 mmol H_3_(BO_3_) and 4.8 g NaOH in 3 mL deionized water. The autoclave was heated to 250 °C at 2 K min^−1^, held for 10 h before being cooled down at a rate of 10 K min^−1^ to room temperature. The reaction products were washed with methanol and stored in air.

### Crystal Structure Determination

SCXRD data were collected in the temperatures range of 100(1)–320(1) K using a Rigaku XtaLAB Synergy‐S diffractometer equipped with a microfocus PhotonJet X‐ray tube source (Mo‐*K*_α_ radiation, *λ*=71.073 pm) and a hybrid photon counting detector (Eiger2 R 1 M CdTe, Dectris). The raw data were processed using CrysAlispro and corrected for background, Lorentz and polarization factors, multi‐scan absorption correction was applied. An initial structural model was obtained by ShelXT.^[63]^ Structure refinement against *F_o_* ^2^ was carried out with ShelXL^[64]^ under Olex2.^[65]^ Anisotropic displacement parameters for all non‐hydrogen atoms. Graphical material of the structures for the publication was rendered with Diamond.^[66]^

Deposition Number(s) 2366122 (for α‐Na_2_[Y(BO_3_)(OH)_2_]), 2366125 (for α‐Na_2_[Gd(BO_3_)(OH)_2_]), 2366126 (for α‐Na_2_[Tb(BO_3_)(OH)_2_]), 2366123 (for α‐Na_2_[Dy(BO_3_)(OH)_2_]), 2364429 (for α‐Na_2_[Ho(BO_3_)(OH)_2_]), 2366127 (for β‐Na_2_[Ho(BO_3_)(OH)_2_]), 2366124 (for α‐Na_2_[Er(BO_3_)(OH)_2_]), 2365879 (for α‐Na_2_[Dy_0.5_Er_0.5_(BO_3_)(OH)_2_]) contain the supplementary crystallographic data for this paper. These data are provided free of charge by the joint Cambridge Crystallographic Data Centre and Fachinformationszentrum Karlsruhe Access Structures service.

### SEM and EDX Analysis

Scanning electron microscopy (SEM) was performed using a SU8020 (Hitachi) with a triple detector system for secondary and low‐energy backscattered electrons (acceleration voltage *U*_a_=5 kV). The composition of selected single crystals was determined by semi‐quantitative energy dispersive X‐ray analysis (*U*_a_=15 kV) using a Silicon Drift Detector (SDD) X–Max^N^ (Oxford Instruments). The data were processed with the AZtec software package (Oxford Instruments, 2013).

### Powder X‐Ray Diffraction

Powder samples were investigated at room temperature on a Stadi P (Stoe & Cie.) diffractometer equipped with a curved Ge‐monochromator and a Dectris Mythen 1 K detector using Cu−Kα_1_ radiation (*λ*=154.056 pm).

### Magnetization Measurement

The magnetic properties between 2 K and 300 K were analyzed with a Cryogen Free Measurement System (CFMS, Cryogenic). The magnetization data were measured using a Vibrating Sample Magnetometer (VSM, 21 Hz) under an external magnetic field of 100 mT or 1 T. Crystal Na_2_[Dy(BO_3_)(OH)_2_] (50.0 mg) and Na_2_[Dy_0.5_Er_0.5_(BO_3_)(OH)_2_] (51.0 mg) samples were sealed in a capsule and attached to a nonmagnetic sample holder.

### Infrared Spectroscopy

IR spectra were recorded using a Bruker Vertex 70 FT‐IR spectrometer. The device was operated in the ATR mode (diamond), working with a measuring range of 500–4000 cm^−1^. Data analysis was performed with the program OPUS.

### Raman and PL Spectroscopy

Raman spectra were measured with three different excitation lasers. In the range of 100–4000 cm^−1^, the 1064, 532 and 458 nm Nd‐YAG excitation laser was used in Bruker RFS 100 Fourier transform Raman spectrometer, Thermo Fischer DXR SmartRaman spectrometer and confocal microscope LabRAM HR Evolution (Horiba). The photoluminescence (PL) spectrum of samples were collected using same microscope with Raman LabRAM HR Evolution (Horiba) under the excitation wavelength of 458 nm in the range of 550–1150 nm.

### UV‐Vis Spectroscopy

The Absorption spectra were recorded with a VARIAN CARY 50 UV/VIS‐ spectrometer equipped with an external diffuse reflectance accessory probe (Barrelino, Harrick Scientific). The powder sample were prepared on a quartz glass placed on BaSO_4_ and measured between 200 and 1000 nm. BaSO_4_ was used as reference for the baseline correction. The sample mass was about 20 mg.

### Thermal Analysis

Na_2_Dy[(BO_3_)(OH)_2_] samples were heated to 1000 °C at a rate of 5 K min^−1^ under a dynamic flow of synthetic air (CO_2_ free) or argon in a STA 409 C/CD (Netzsch). The phase transition depends on temperature was studied using a DSC1 from Mettler Toledo. The DSC experiments were carried out in nitrogen atmosphere between 60 and −15 °C applying a cooling and heating rate of 1 K min^−1^. Firstly cooling to −15 °C and then, heated back for the second cooling with the same rate.

## Conflict of Interests

The authors declare no conflict of interest.

1

## Supporting information

As a service to our authors and readers, this journal provides supporting information supplied by the authors. Such materials are peer reviewed and may be re‐organized for online delivery, but are not copy‐edited or typeset. Technical support issues arising from supporting information (other than missing files) should be addressed to the authors.

Supporting Information

## Data Availability

The data that support the findings of this study are available from the corresponding author upon reasonable request.
