# Supplementary material for: Hydroflux Synthesis and Structural Phase Transition of Rare‐Earth Borate Hydroxides Na2[RE(BO3)(OH)2] (RE=Y, Gd−Er)
Source: Chemistry. 2024 Oct 16;30(68):e202402783. doi: 10.1002/chem.202402783 (PMC11618041; doi:10.1002/chem.202402783)
Supplement: Supplementary file 1 — Supporting Information [file CHEM-30-e202402783-s001.pdf]

Chemistry–A European Journal

Supporting Information

Hydroflux Synthesis and Structural Phase Transition of Rare-Earth Borate Hydroxides $Na_2[RE(BO_3)(OH)_2]$ (RE = Y, Gd–Er)

Yuxi Li, Eduardo Carrillo-Aravena, Jiang Qu, Gohil S. Thakur, and Michael Ruck*

CONTENTS

Figure S1. Photographs of $Na_2[RE(BO_3)(OH)_2]$ (*RE*= Y, Gd – Dy, Er) crystals. 5

Figure S2. SEM image of $Na_2[Ho(BO_3)(OH)_2]$ crystal washed with water (a, b) or methanol (c, d), and after two months in air (d). 5

Figure S3. PXRD patterns of $Na_2[RE(BO_3)(OH)_2]$ (*RE* = Y, Gd – Er) at 296 K. 6

Figure S4. PXRD patterns of $Na_2[Ho(BO_3)(OH)_2]$. 6

Figure S5. Lattice parameters and unit cell volumes of $Na_2[RE(BO_3)(OH)_2]$ (*RE* = Y, Gd – Er) against the ionic radius of RE^{3+} cations in seven-coordination. The lattice parameters were taken from single-crystal diffraction data at 100K. 7

Figure S6. SEM image of $Na_2[Dy_{0.5}Er_{0.5}(BO_3)(OH)_2]$ crystal and EDX mappings. 7

Figure S7. PXRD patterns and Rietveld refinement of $Na_2[Dy_{0.5}Er_{0.5}(BO_3)(OH)_2]$ in the space group *C*2/*c*. 8

Figure S8. Structures of $Na_2[Ho(BO_3)(OH)_2]$ at 310 K and 100 K projected along the *a* and *c* axes. 8

Figure S9. (a) DyO_7 polyhedron and (b) $(BO_3)^{3-}$ anion in $Na_2[Dy(BO_3)(OH)_2]$. The ellipsoids enclose a space in which with 90% probability the electron density of the atoms can be found at 100 K. 9

Figure S10. Variation on the lattice parameters of $Na_2[Ho(BO_3)(OH)_2]$ as a function of temperature. The error bars represent ±3 standard deviations. 9

Figure S11. Group-subgroup relation between orthorhombic β- and monoclinic α-$Na_2[Ho(BO_3)(OH)_2]$ in a Bärnighausen tree. The monoclinic space group *C* 1 2/*c* 1 is a maximum translationengleiche subgroup of index 2 of the orthorhombic space group *C* 2/*m* 2/*c* 2_1/*m*. The transition from *Cmcm* to *C*2/*c* includes no change of axes, no origin shift, and no splitting of the occupied atomic positions. The transcribed atomic positions are given in normal fonts, the experimentally found ones in italics. 10

Figure S12. Temperature dependence of possible order parameters during the phase transition of $Na_2[Ho(BO_3)(OH)_2]$: $\eta = \beta - \beta_c$ using the monoclinic angle (left top), $\eta = ((x - x_c)^2 + (y - y_c)^2 + (z - z_c)^2)^{1/2}$ using the Na atom (right top), the O1 atom (left bottom) or the O2 atom (right bottom). The error bars show ±3 standard deviations. The estimated error in the temperature is ±1 K. ... 11

Figure S13. DSC curves of selected crystals of $Na_2[Ho(BO_3)(OH)_2]$ between −15 °C and +60 °C, cooling and heating at 5 K min^{-1} (open crucible in nitrogen atmosphere). 12

Figure S14. Thermal analysis of $Na_2[Dy(BO_3)(OH)_2]$ in synthetic argon with a heating rate of 5 K·min^{-1}. 12

Figure S15. PXRD pattern of the thermal decomposition product of $Na_2[Dy(BO_3)(OH)_2]$ together with the reference patterns of $Na_3Dy(BO_3)_2$ and Dy_2O_3. 13

Figure S16. SEM images of $Na_2[Dy(BO_3)(OH)_2]$ after annealing in air at 500 °C. 13

Figure S17. Images of $Na_2[Dy(BO_3)(OH)_2]$ and $Na_2[Dy(BO_3)(OH)_2]$ annealed sample were placed on the pH test paper in air for 2 hours. 14

Figure S18. Raman spectrum of $Na_2[RE(BO_3)(OH)_2]$ (*RE* = Y, Gd – Er) measured at room temperature at different laser Wavelength 458 nm (a), 532 nm (b). 14

Figure S19. UV/Vis diffuse reflectance spectrum of $Na_2[\mathbf{Y}(BO_3)(OH)_2]$ (a) and Tauc plots for indirect (b) and direct (c) optical transitions. 15

Figure S20. UV/Vis diffuse reflectance spectrum of $Na_2[\mathbf{Gd}(BO_3)(OH)_2]$ (a) and Tauc plots for indirect (b) and direct (c) optical transitions. 15

Figure S22. UV/Vis diffuse reflectance spectrum of $Na_2[\mathbf{Ho}(BO_3)(OH)_2]$ (a) and Tauc plots for indirect (b) and direct (c) optical transitions. 16

Figure S23. UV/Vis diffuse reflectance spectrum of $Na_2[\mathbf{Er}(BO_3)(OH)_2]$ (a) and Tauc plots for indirect (b) and direct (c) optical transitions. 17

Figure S24. Molar magnetic susceptibility and its inverse for $Na_2[Dy_{0.5}Er_{0.5}(BO_3)(OH)_2]$ measured in a field of $\mu_0 H$ = 0.1 T between 2 and 300 K. The blue line is the linear Curie-Weiss fit. 17

Figure S25. X-ray diffraction patterns *h*0*l* of $Na_2[RE(BO_3)(OH)_2]$ (*RE* = Y, Gd–Er) at 100 K. All crystals are twinned along [100], but the crystals of the Gd and Dy compounds are also twinned along [001]. The patterns of *RE* = Tb, Dy, Er show weak reflections that violate the reflection condition of the *c* glide plane (*h*0*l* only with *l* = 2*n*). As the structural models show no anomalies, the space group *C*2/*c* was retained as a (very good) approximation. 18

Table S1. EDX-derived compositions of the rare earth compounds $Na_2[Dy_{0.5}Er_{0.5}(BO_3)(OH)_2]$. The given standard deviations are based on the statistics of multiple measurements but do not include systematic errors. 19

Table S2. Crystallographic data for $Na_2[RE(BO_3)(OH)_2]$ (*RE* = Y, Gd–Er) at 100 K. All crystals are twinned along [100], but the crystals of the Gd and Dy compounds are also twinned along [001] (Figure S25). Refinements based on HKLF5 data have been exclusively performed for the twin along [100]. 19

Table S3. Crystallographic data for $Na_2[Ho(BO_3)(OH)_2]$ at 310 K. 20

Table S4. Monoclinic angles (space group *C*2/*c*) of $Na_2[RE(BO_3)(OH)_2]$ (*RE* = Y, Gd–Er) at room temperature (296 K). 20

Table S5. Space group (SG), lattice parameters (/pm), β-angle (/°) and unit cell volumes (/10^6 pm^3) of Na_2**Ho**[$(BO_3)(OH)_2$] at different temperatures. 21

Table S6. Coordinates and coefficients U_{ij} (/ pm^2) of the tensor of the anisotropic displacement factor and equivalent or isotropic displacement factor for the atoms in β-Na_2[**Ho**$(BO_3)(OH)_2$] at 310 K (space group ***Cmcm***). U_{eq} is defined as one third of the trace of the orthogonalized U_{ij} tensor. All atoms occupy general Wyckoff positions. 21

Table S7. Coordinates and coefficients U_{ij} (/ pm^2) of the tensor of the anisotropic displacement factor and equivalent or isotropic displacement factor for the atoms in α-Na_2[**Y**$(BO_3)(OH)_2$] at 100 K (space group ***C2/c***). U_{eq} is defined as one third of the trace of the orthogonalized U_{ij} tensor. All atoms occupy general Wyckoff positions. 21

Table S8. Coordinates and coefficients U_{ij} (/ pm^2) of the tensor of the anisotropic displacement factor and equivalent or isotropic displacement factor for the atoms in α-Na_2[**Gd**$(BO_3)(OH)_2$] at 100 K (space group ***C2/c***). U_{eq} is defined as one third of the trace of the orthogonalized U_{ij} tensor. All atoms occupy general Wyckoff positions. 22

Table S9. Coordinates and coefficients U_{ij} (/ pm^2) of the tensor of the anisotropic displacement factor and equivalent or isotropic displacement factor for the atoms in α-Na_2[**Tb**$(BO_3)(OH)_2$] at 100 K (space group ***C2/c***). U_{eq} is defined as one third of the trace of the orthogonalized U_{ij} tensor. All atoms occupy general Wyckoff positions. 22

Table S10. Coordinates and coefficients U_{ij} (/ pm^2) of the tensor of the anisotropic displacement factor and equivalent or isotropic displacement factor for the atoms in α-Na_2[**Dy**$(BO_3)(OH)_2$] at 100 K (space group ***C2/c***). U_{eq} is defined as one third of the trace of the orthogonalized U_{ij} tensor. All atoms occupy general Wyckoff positions. 22

Table S11. Coordinates and coefficients U_{ij} (/ pm^2) of the tensor of the anisotropic displacement factor and equivalent or isotropic displacement factor for the atoms in α-Na_2[**Ho**$(BO_3)(OH)_2$] at 100 K (space group ***C2/c***). U_{eq} is defined as one third of the trace of the orthogonalized U_{ij} tensor. All atoms occupy general Wyckoff positions. 23

Table S12. Coordinates and coefficients U_{ij} (/ pm^2) of the tensor of the anisotropic displacement factor and equivalent or isotropic displacement factor for the atoms in α-Na_2[**Er**$(BO_3)(OH)_2$] at 100 K (space group ***C2/c***). U_{eq} is defined as one third of the trace of the orthogonalized U_{ij} tensor. All atoms occupy general Wyckoff positions. 23

Table S13. Coordinates and coefficients U_{ij} (/ pm^2) of the tensor of the anisotropic displacement factor and equivalent or isotropic displacement factor for the atoms in α-Na_2[**$Dy_{0.5}Er_{0.5}$**$(BO_3)(OH)_2$] at 100 K (space group ***C2/c***). U_{eq} is defined as one third of the trace of the orthogonalized U_{ij} tensor. All atoms occupy general Wyckoff positions. 23

Table S14. Comparison of the coordinates and coefficients U_{ij} (/ pm^2) of the tensor of the anisotropic displacement factor and equivalent or isotropic displacement factor for the atoms in α-Na_2[**Ho**$(BO_3)(OH)_2$] at 100 K,150 K, 200 K, 250 K, 270 K, 280 K, 290 K, 300 K. All atoms occupy general Wyckoff positions. 24

Table S15. Comparison of the coordinates and coefficients U_{ij} (/ pm^2) of the tensor of the anisotropic displacement factor and equivalent or isotropic displacement factor for the atoms in Na_2[**Ho**$(BO_3)(OH)_2$] at 310 K and 320 K. All atoms occupy general Wyckoff positions. 25

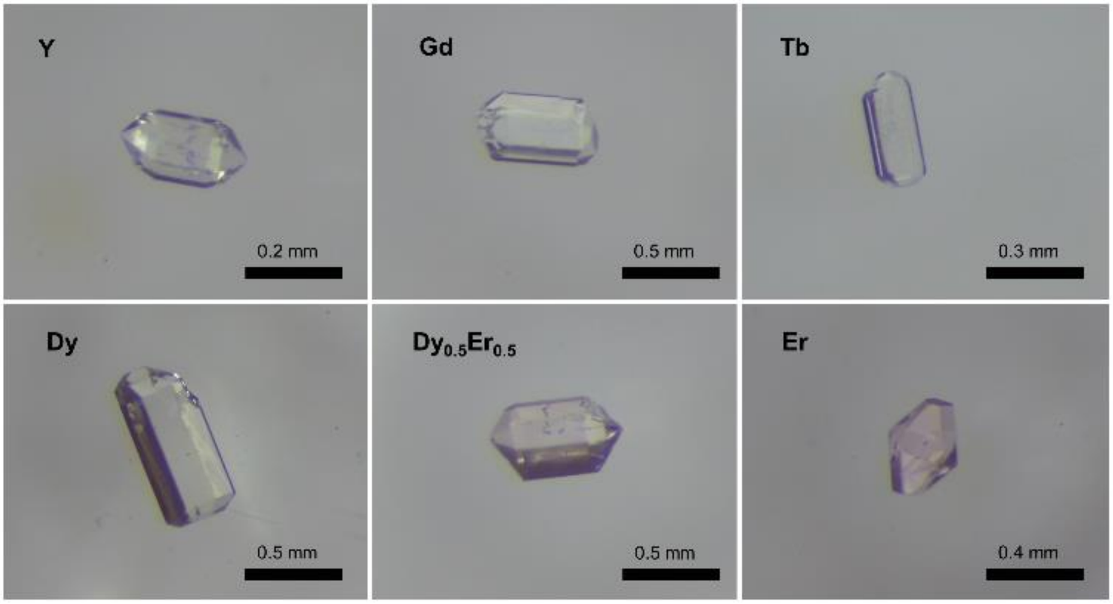

Figure S1. Photographs of $Na_2[RE(BO_3)(OH)_2]$ (*RE*= Y, Gd – Dy, Er) crystals.

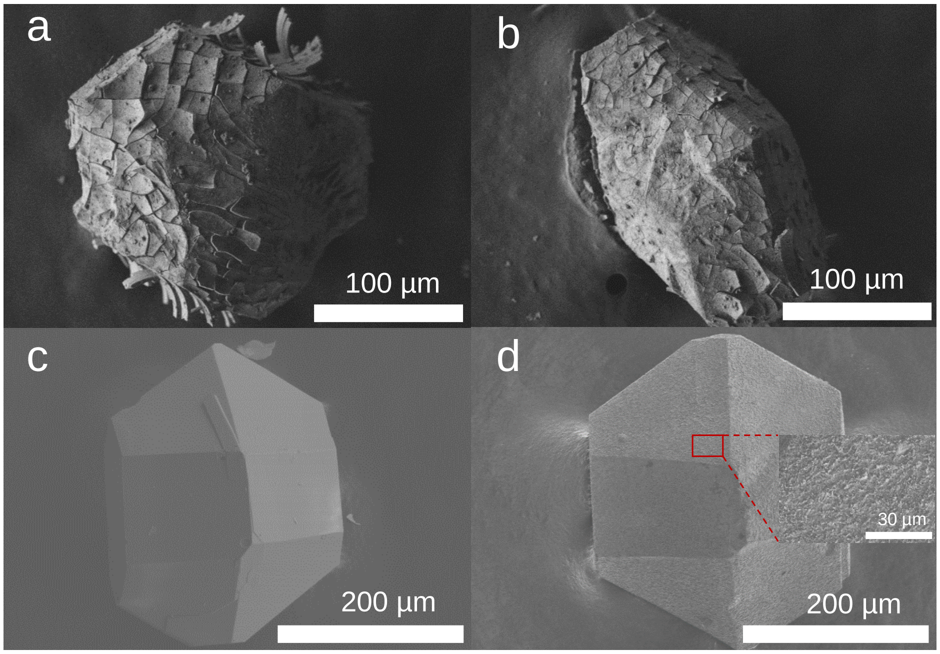

Figure S2. SEM image of $Na_2[Ho(BO_3)(OH)_2]$ crystal washed with water (a, b) or methanol (c, d), and after two months in air (d).

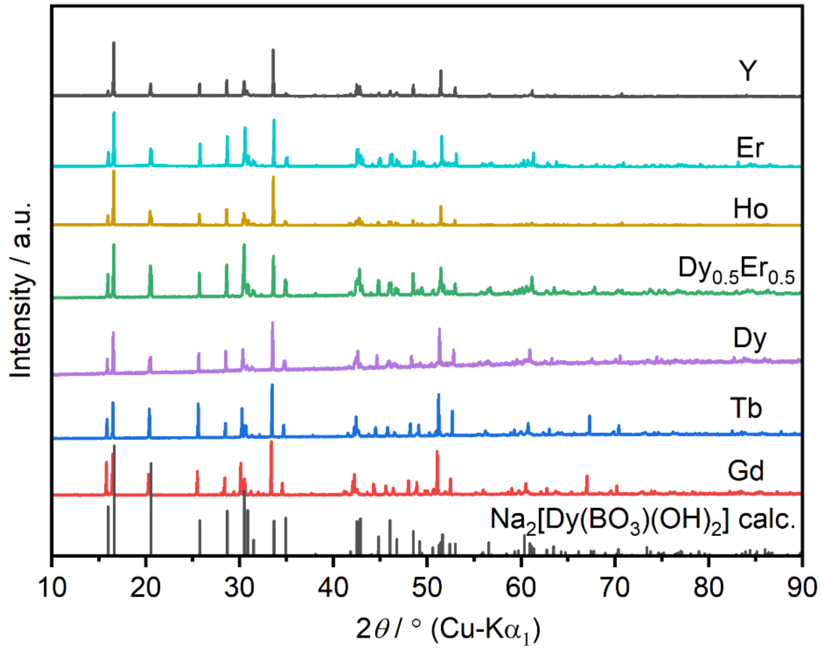

Figure S3. PXRD patterns of $Na_2[\mathit{RE}(BO_3)(OH)_2]$ (*RE* = Y, Gd – Er) at 296 K.

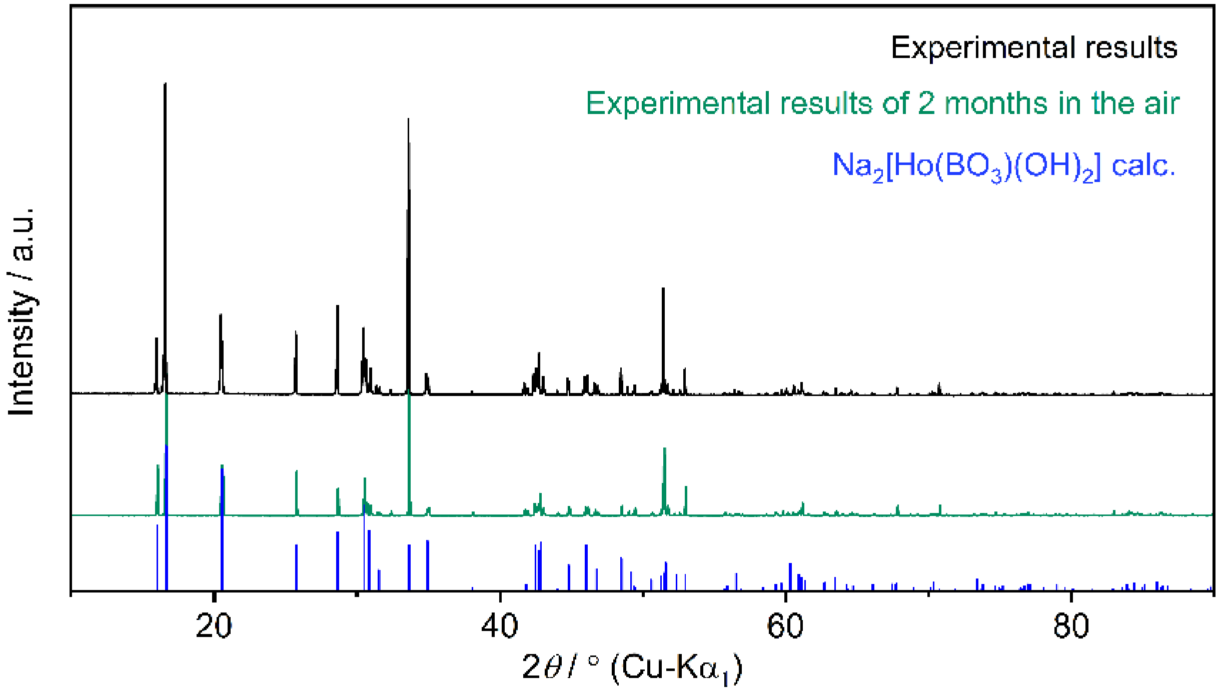

Figure S4. PXRD patterns of $Na_2[Ho(BO_3)(OH)_2]$.

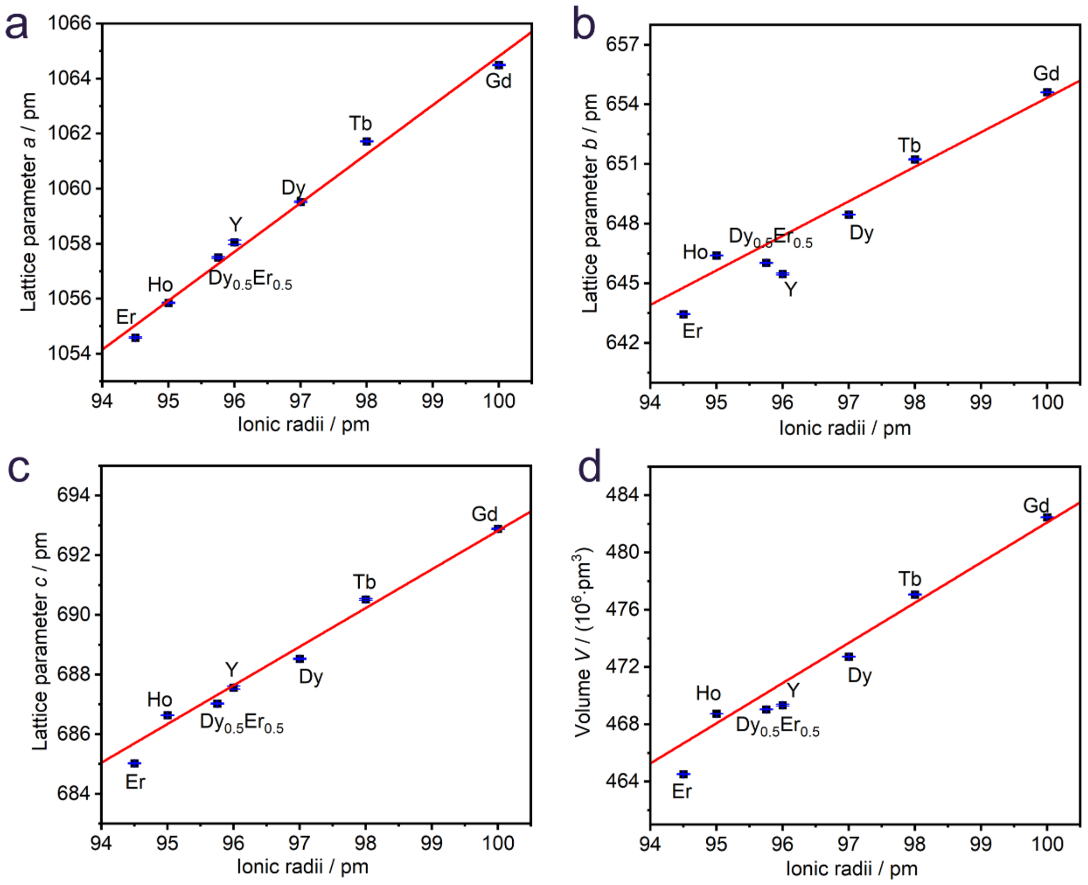

Figure S5. Lattice parameters and unit cell volumes of $Na_2[RE(BO_3)(OH)_2]$ (*RE* = Y, Gd – Er) against the ionic radius of RE^{3+} cations in seven-coordination. The lattice parameters were taken from single-crystal diffraction data at 100K.

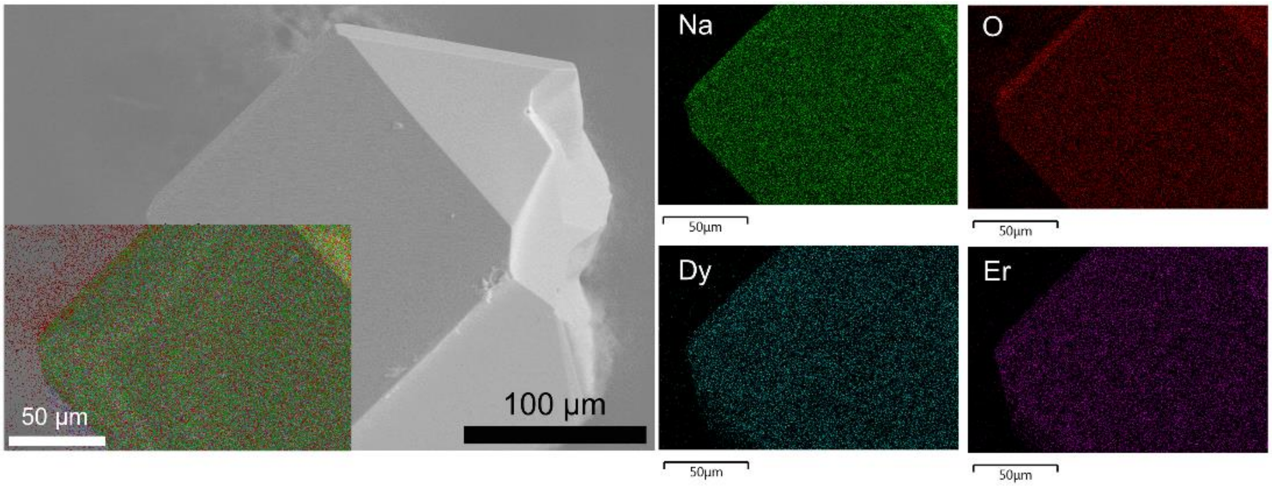

Figure S6. SEM image of $Na_2[Dy_{0.5}Er_{0.5}(BO_3)(OH)_2]$ crystal and EDX mappings.

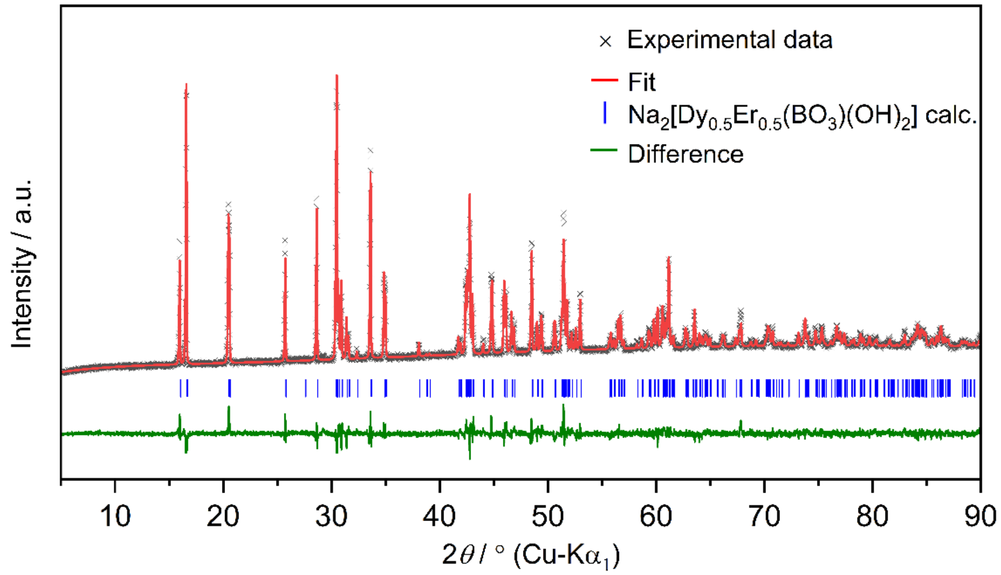

Figure S7. PXRD patterns and Rietveld refinement of $Na_2[Dy_{0.5}Er_{0.5}(BO_3)(OH)_2]$ in the space group *C*2/*c*.

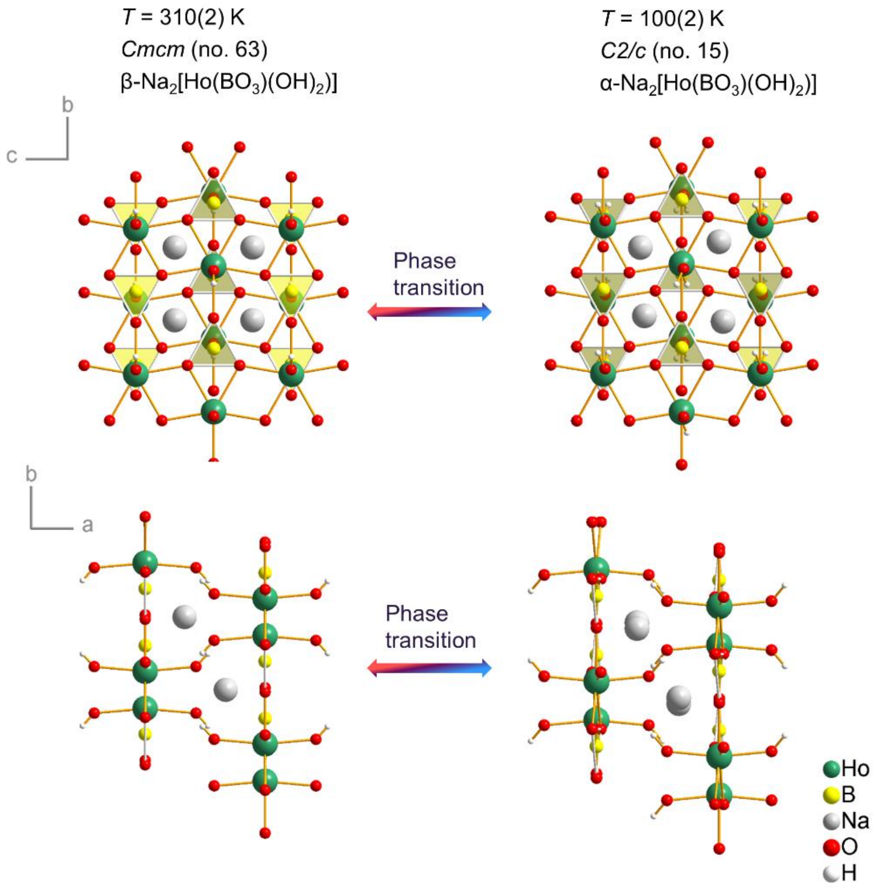

Figure S8. Structures of $Na_2[Ho(BO_3)(OH)_2]$ at 310 K and 100 K projected along the *a* and *c* axes.

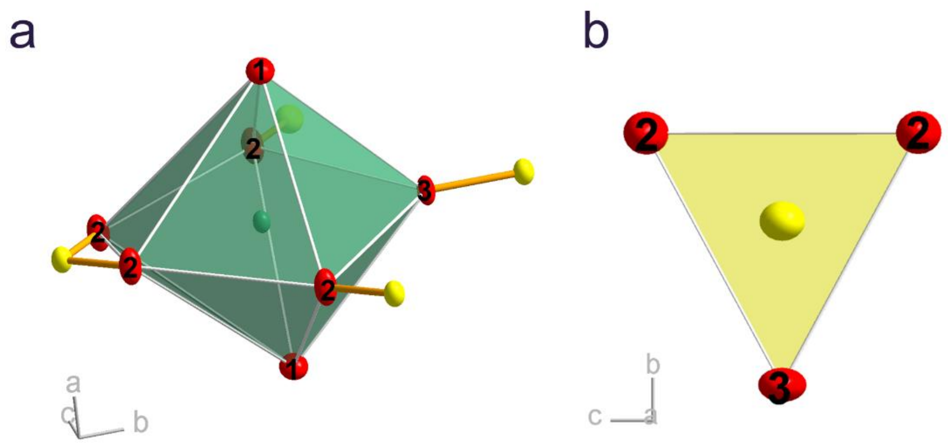

Figure S9. (a) DyO_7 polyhedron and (b) $(BO_3)^{3-}$ anion in $Na_2[Dy(BO_3)(OH)_2]$. The ellipsoids enclose a space in which with 90% probability the electron density of the atoms can be found at 100 K.

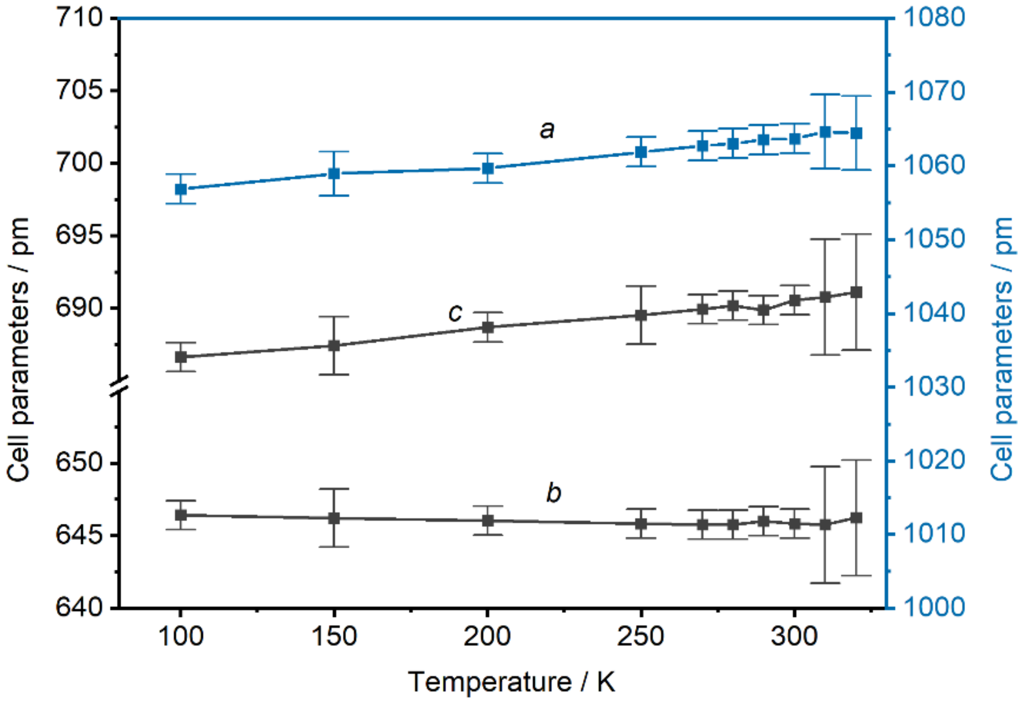

Figure S10. Variation on the lattice parameters of $Na_2[Ho(BO_3)(OH)_2]$ as a function of temperature. The error bars represent ±3 standard deviations.

T = 310(2) K

Cmcm (no. 63)
*C*2/*m*2/*c*2$_1$/*m* (no. 63)
β-$Na_2[Ho(BO_3)(OH)_2)]$

Ho: 4*c*	Na: 8*e*	B: 4*c*	O1: 8*g*	O2: 8*f*	O3: 4*c*	H: 8*g*
m 2m	2	m 2m	..m	m..	m 2m	..m
0	0.164	0	0.213	0	0	0.259
0.129	½	0.693	0.160	0.190	0.484	0.287
¼	0	¼	¼	0.582	¼	¼

t2

Ho: 4*e*	Na: 8*f*	B: 4*e*	O1: 8*f*	O2: 8*f*	O3: 4*e*	H: 8*f*
2	1	2	1	1	2	1
0	0.164	0	0.213	0	0	0.259
0.129	½	0.693	0.160	0.190	0.484	0.287
¼	0	¼	¼	0.582	¼	¼

T = 100(2) K

*C*2/*c* (no. 15)
*C*12/*c*1 (no. 15)
α-$Na_2[Ho(BO_3)(OH)_2)]$

Ho: 4*e*	Na: 8*f*	B: 4*e*	O1: 8*f*	O2: 8*f*	O3: 4*e*	H: 8*f*
2	1	2	1	1	2	1
0	*0.165*	*0*	*0.213*	*0.018*	*0*	*0.262*
0.130	*0.481*	*0.697*	*0.163*	*0.189*	*0.486*	*0.270*
¼	*0.013*	*¼*	*0.237*	*0.583*	*¼*	*0.285*

Figure S11. Group-subgroup relation between orthorhombic β- and monoclinic α-$Na_2[Ho(BO_3)(OH)_2]$ in a Bärnighausen tree. The monoclinic space group *C* 1 2/*c* 1 is a maximum translationengleiche subgroup of index 2 of the orthorhombic space group *C* 2/*m* 2/*c* 2$_1$/*m*. The transition from *Cmcm* to *C*2/*c* includes no change of axes, no origin shift, and no splitting of the occupied atomic positions. The transcribed atomic positions are given in normal fonts, the experimentally found ones in italics.

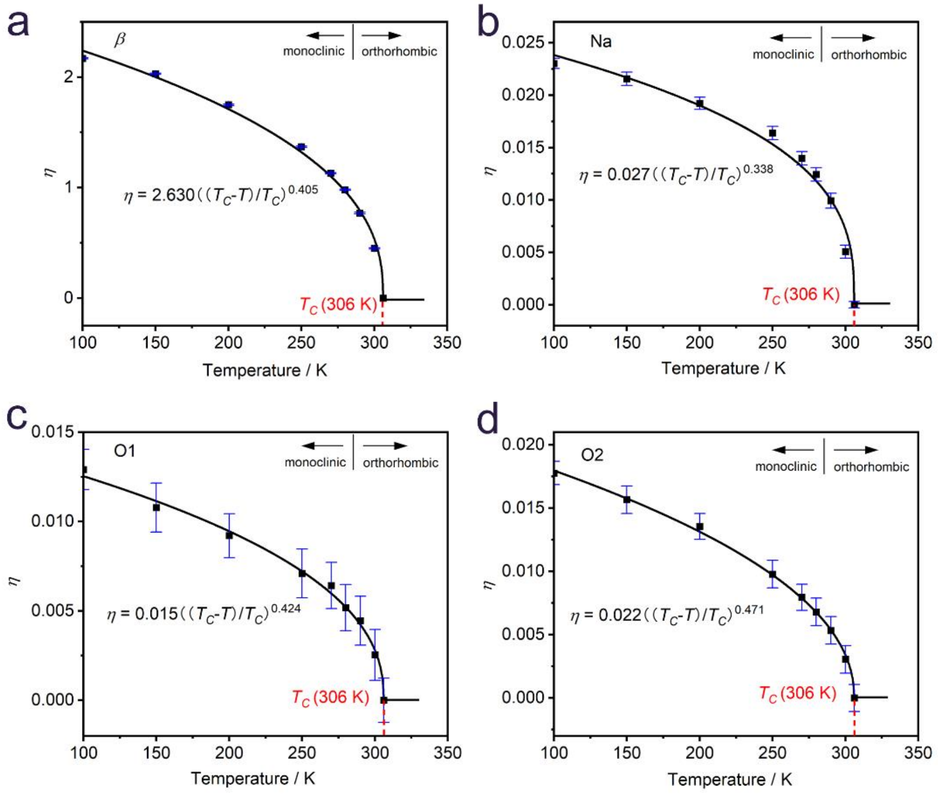

Figure S12. Temperature dependence of possible order parameters during the phase transition of $Na_2[Ho(BO_3)(OH)_2]$: $\eta = \beta - \beta_c$ using the monoclinic angle (left top), $\eta = ((x - x_c)^2 + (y - y_c)^2 + (z - z_c)^2)^{1/2}$ using the Na atom (right top), the O1 atom (left bottom) or the O2 atom (right bottom). The error bars show ±3 standard deviations. The estimated error in the temperature is ±1 K.

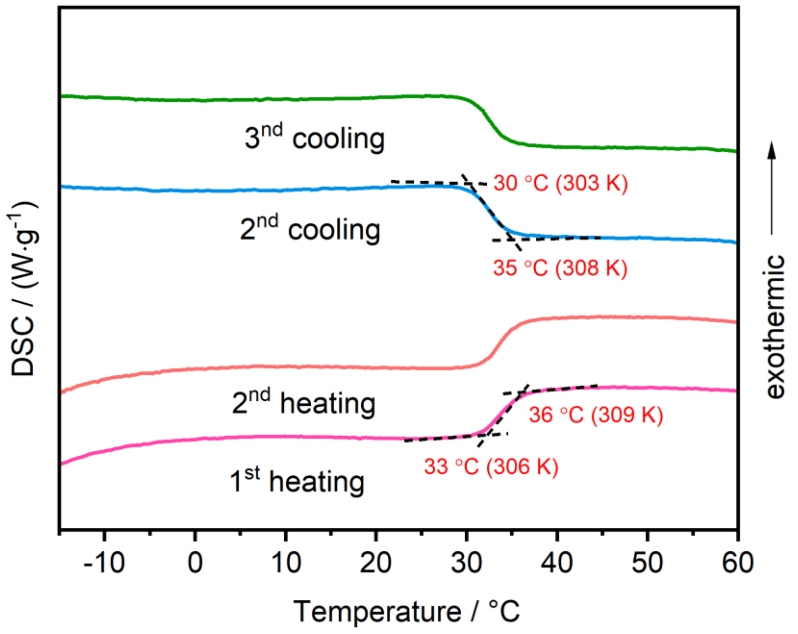

Figure S13. DSC curves of selected crystals of $Na_2[Ho(BO_3)(OH)_2]$ between −15 °C and +60 °C, cooling and heating at 5 K min^{-1} (open crucible in nitrogen atmosphere).

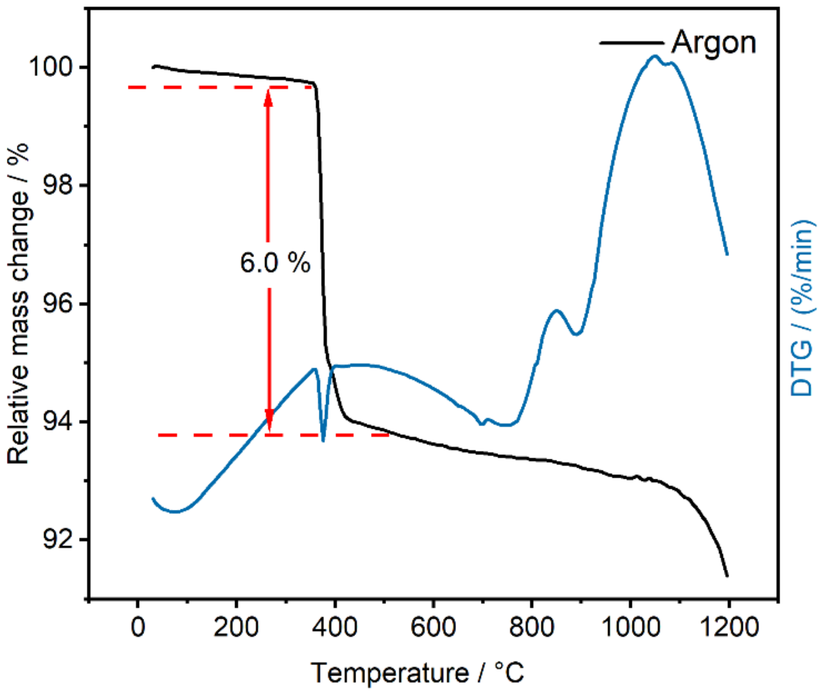

Figure S14. Thermal analysis of $Na_2[Dy(BO_3)(OH)_2]$ in synthetic argon with a heating rate of 5 K·min^{-1}.

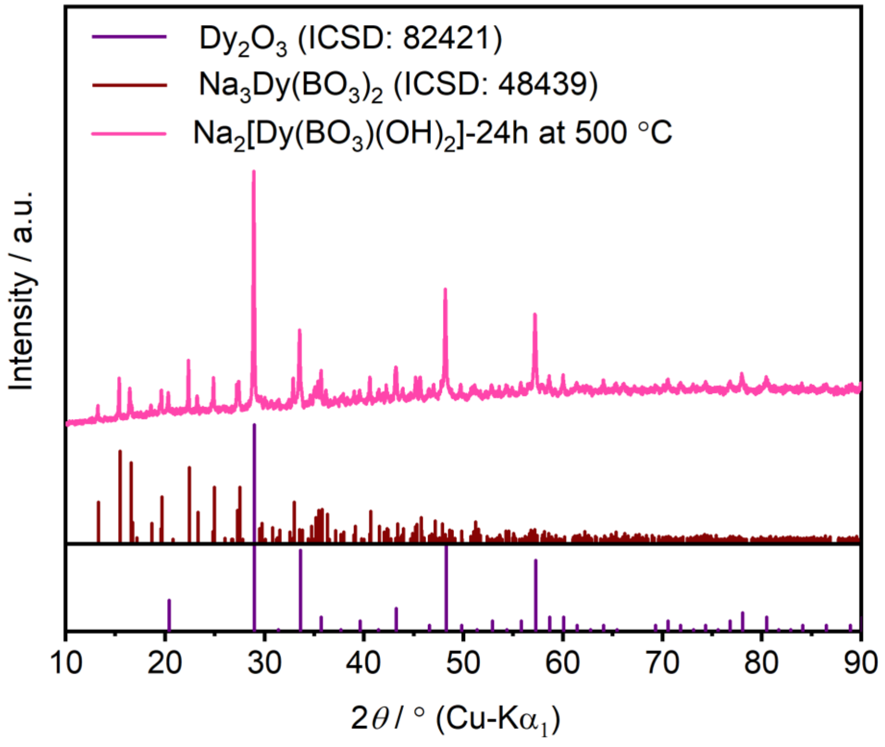

Figure S15. PXRD pattern of the thermal decomposition product of $Na_2[Dy(BO_3)(OH)_2]$ together with the reference patterns of $Na_3Dy(BO_3)_2$ and Dy_2O_3.

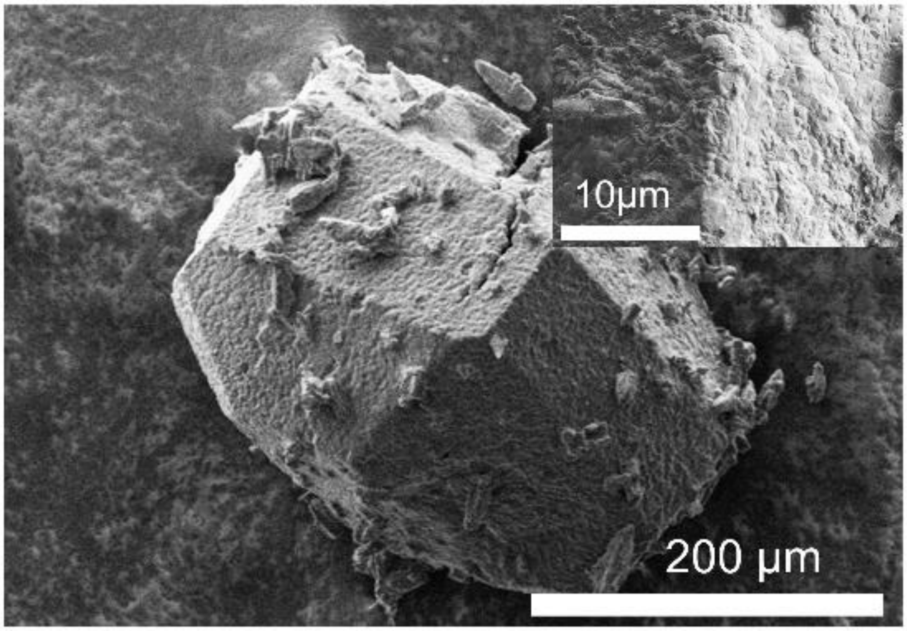

Figure S16. SEM images of $Na_2[Dy(BO_3)(OH)_2]$ after annealing in air at 500 °C.

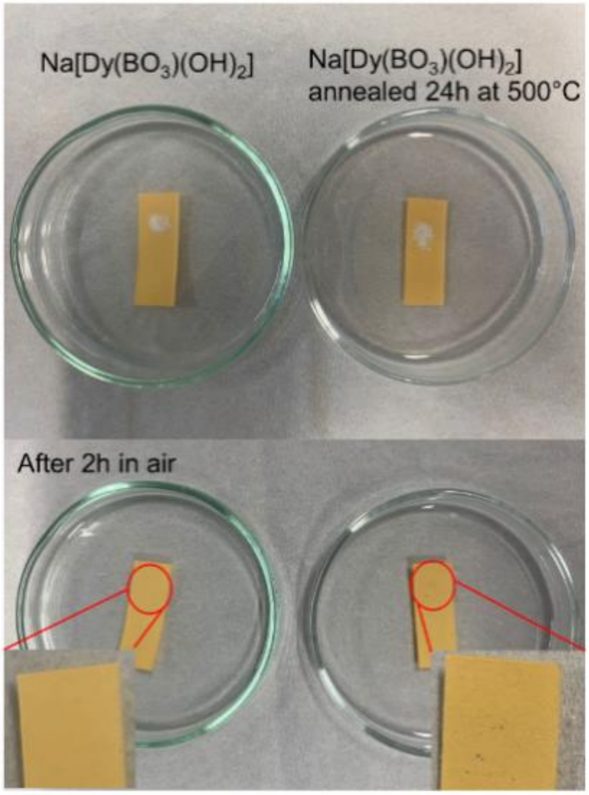

Figure S17. Images of $Na_2[Dy(BO_3)(OH)_2]$ and $Na_2[Dy(BO_3)(OH)_2]$ annealed sample were placed on the pH test paper in air for 2 hours.

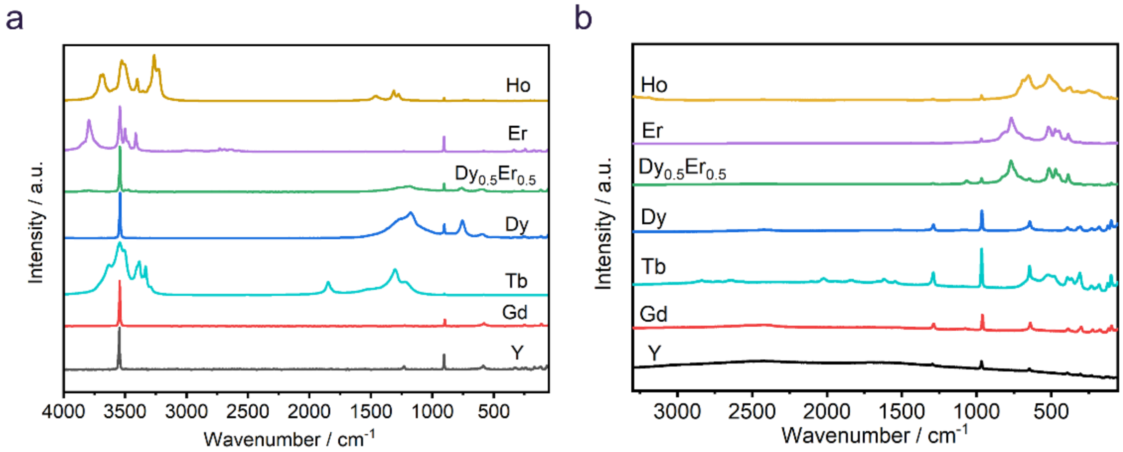

Figure S18. Raman spectrum of $Na_2[RE(BO_3)(OH)_2]$ (*RE* = Y, Gd – Er) measured at room temperature at different laser Wavelength 458 nm (a), 532 nm (b).

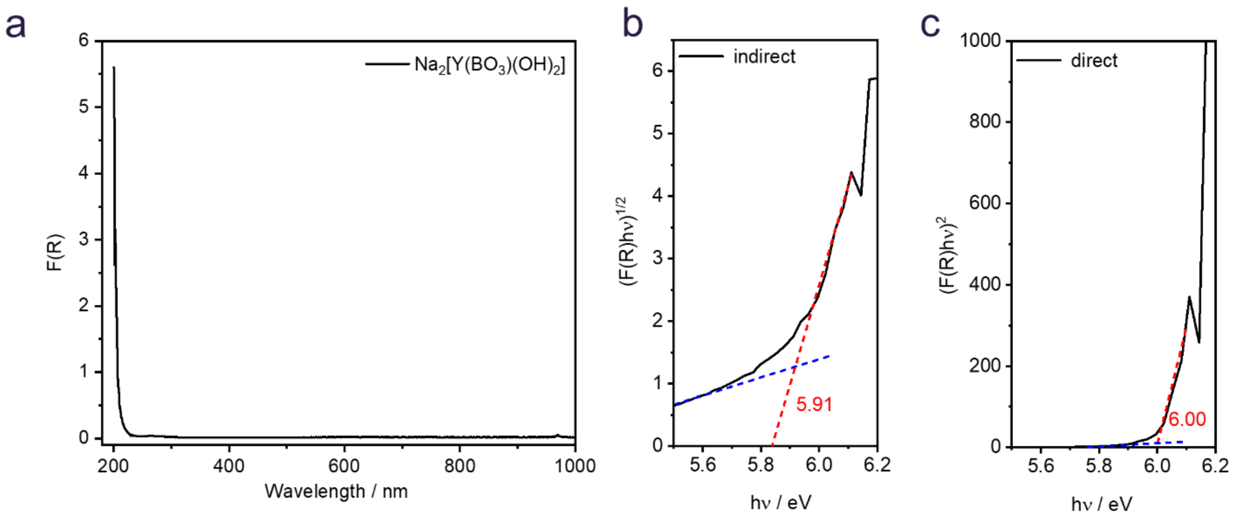

Figure S19. UV/Vis diffuse reflectance spectrum of Na_2[**Y**(BO_3)$(OH)_2$] (a) and Tauc plots for indirect (b) and direct (c) optical transitions.

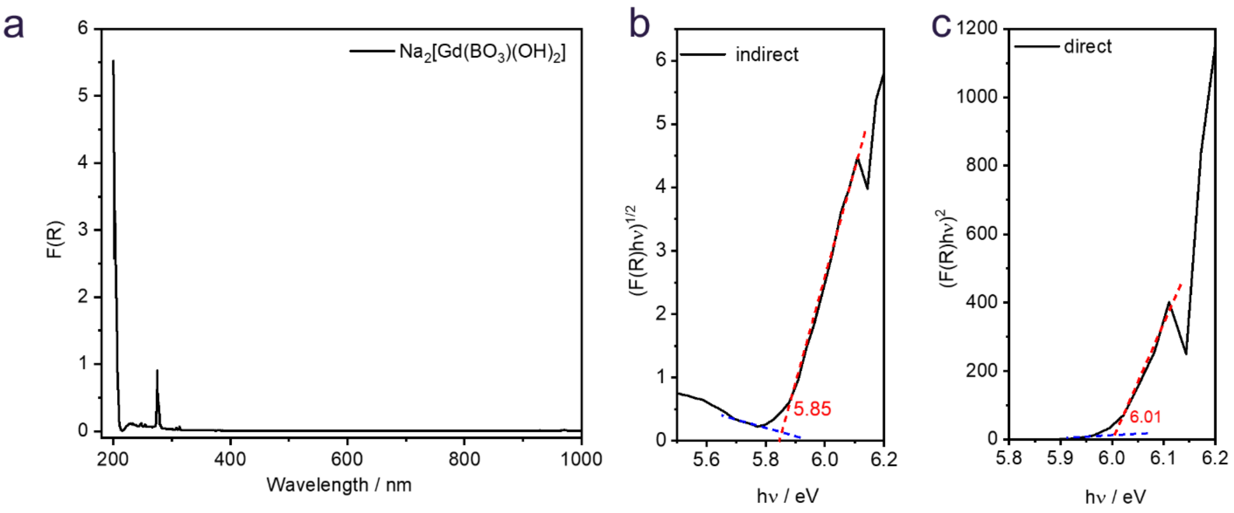

Figure S20. UV/Vis diffuse reflectance spectrum of Na_2[**Gd**(BO_3)$(OH)_2$] (a) and Tauc plots for indirect (b) and direct (c) optical transitions.

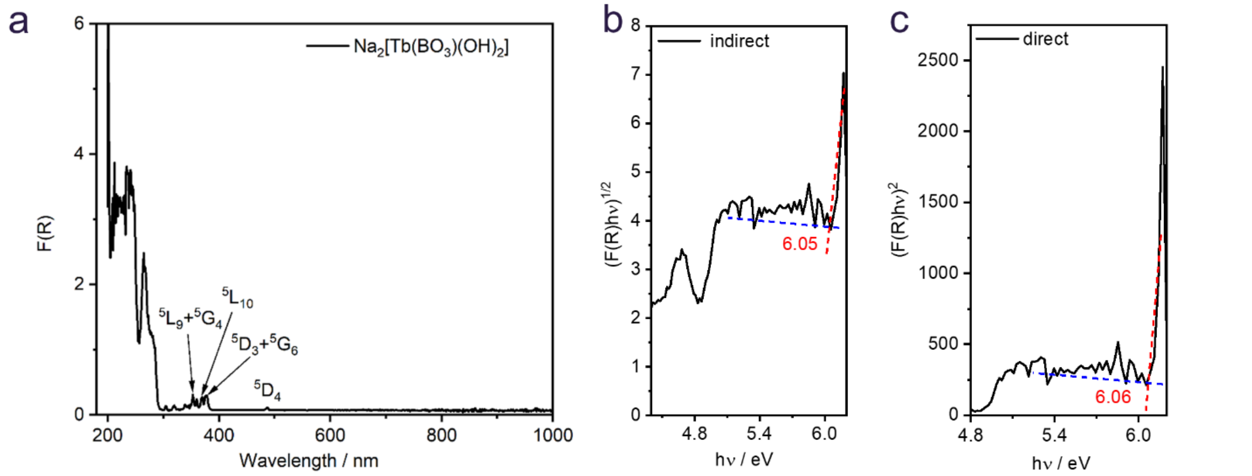

Figure S21. UV/Vis diffuse reflectance spectrum of $Na_2[\mathbf{Tb}(BO_3)(OH)_2]$ (a) and Tauc plots for indirect (b) and direct (c) optical transitions.

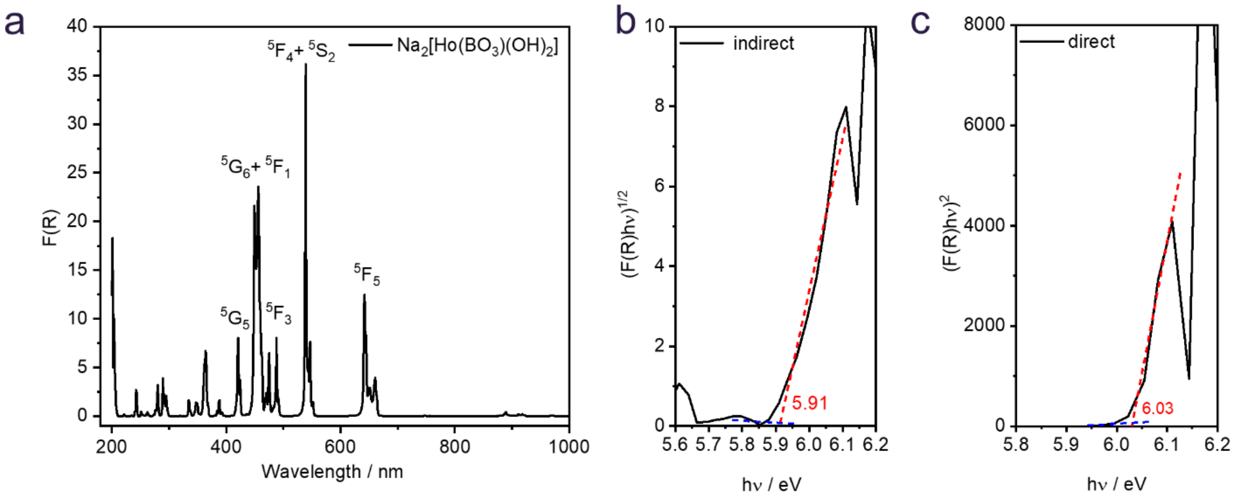

Figure S22. UV/Vis diffuse reflectance spectrum of $Na_2[\mathbf{Ho}(BO_3)(OH)_2]$ (a) and Tauc plots for indirect (b) and direct (c) optical transitions.

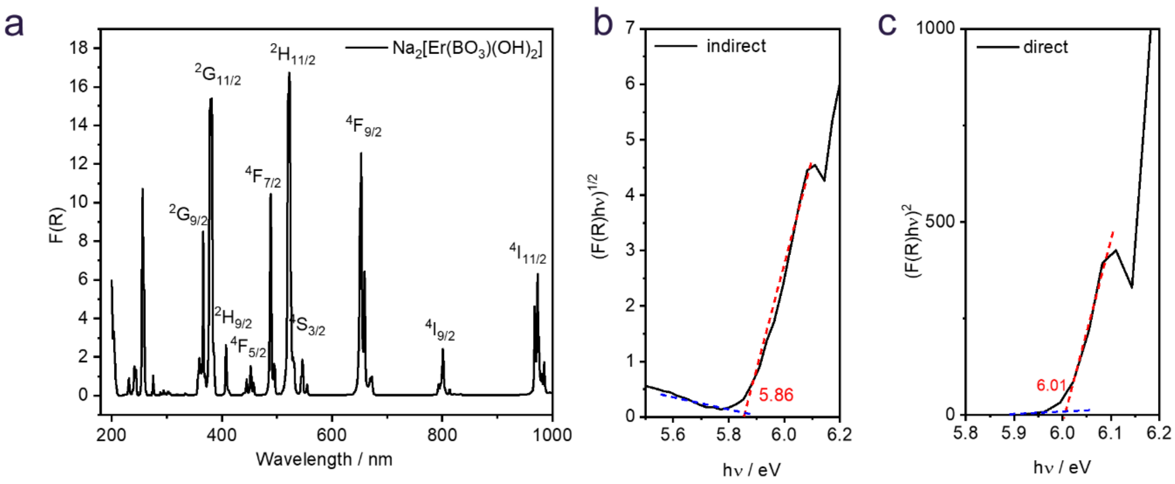

Figure S23. UV/Vis diffuse reflectance spectrum of Na_2[**Er**$(BO_3)(OH)_2$] (a) and Tauc plots for indirect (b) and direct (c) optical transitions.

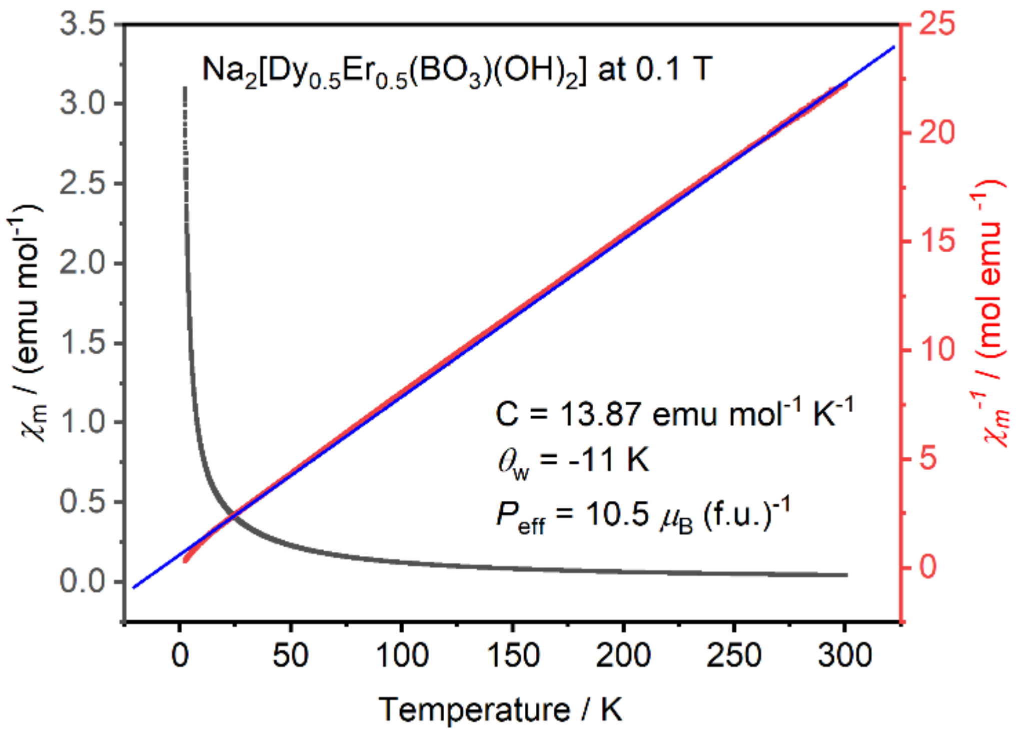

Figure S24. Molar magnetic susceptibility and its inverse for $Na_2[Dy_{0.5}Er_{0.5}(BO_3)(OH)_2]$ measured in a field of $\mu_0 H$ = 0.1 T between 2 and 300 K. The blue line is the linear Curie-Weiss fit.

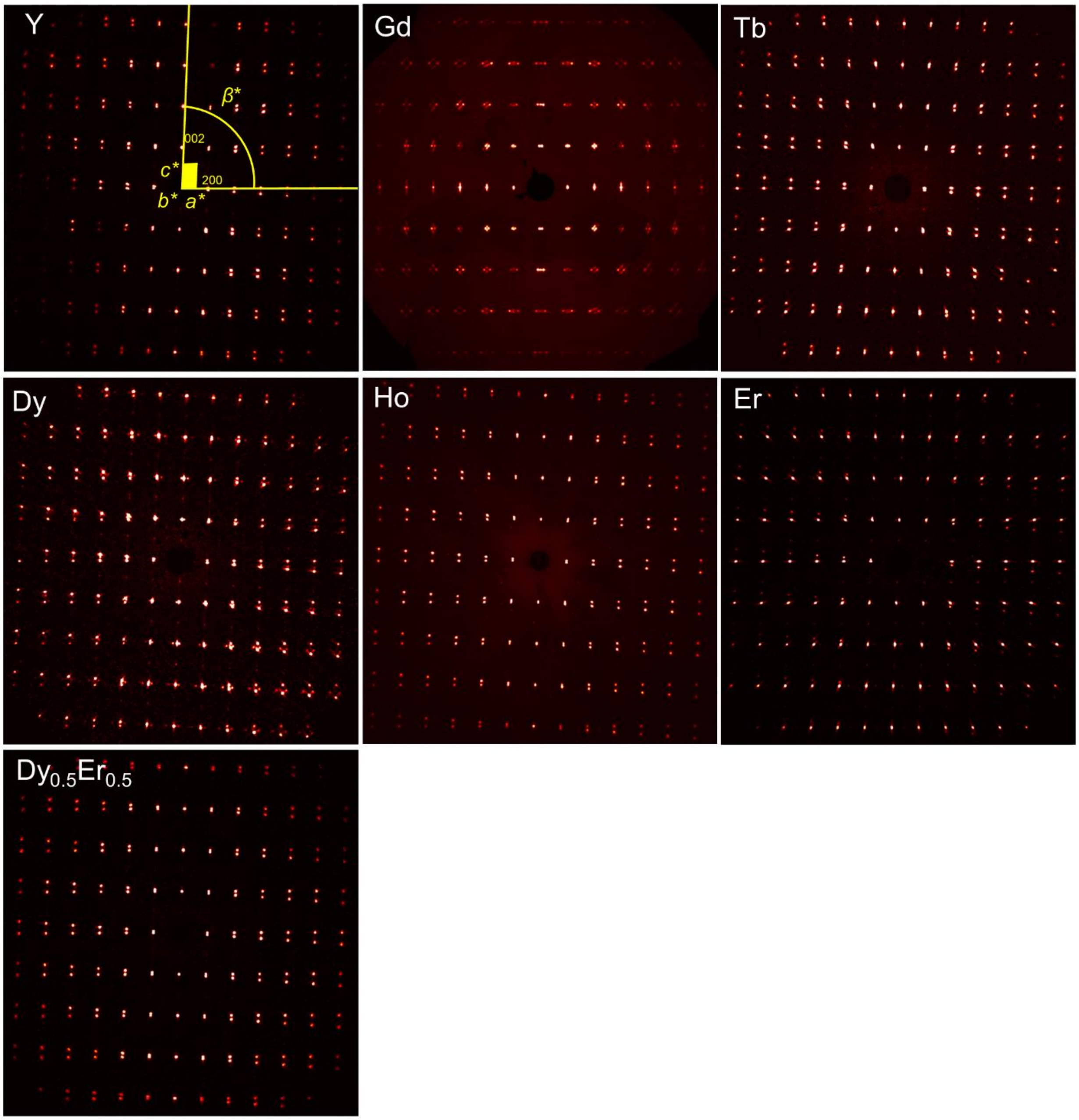

Figure S25. X-ray diffraction patterns *h*0*l* of $Na_2[RE(BO_3)(OH)_2]$ (*RE* = Y, Gd–Er) at 100 K. All crystals are twinned along [100], but the crystals of the Gd and Dy compounds are also twinned along [001]. The patterns of *RE* = Tb, Dy, Er show weak reflections that violate the reflection condition of the *c* glide plane (*h*0*l* only with *l* = 2*n*). As the structural models show no anomalies, the space group *C*2/*c* was retained as a (very good) approximation.

Table S1. EDX-derived compositions of the rare earth compounds $Na_2[Dy_{0.5}Er_{0.5}(BO_3)(OH)_2]$. The given standard deviations are based on the statistics of multiple measurements but do not include systematic errors.

Compound	Na	Er	Dy	B	O
theoretical	4	1	1	2	10
$Na_2[Dy_{0.5}Er_{0.5}(BO_3)(OH)_2]$	3.5(2)	0.84(4)	1.00(5)	2.65(4)	11.2(4)

Table S2. Crystallographic data for $Na_2[RE(BO_3)(OH)_2]$ (*RE* = Y, Gd–Er) at 100 K. All crystals are twinned along [100], but the crystals of the Gd and Dy compounds are also twinned along [001] (Figure S25). Refinements based on HKLF5 data have been exclusively performed for the twin along [100].

$Na_2RE[(BO_3)(OH)_2]$	Y	Gd	Tb	Dy	Ho	Er	$Dy_{0.5}Er_{0.5}$
Crystal system	monoclinic	monoclinic	monoclinic	monoclinic	monoclinic	monoclinic	monoclinic
Space group	*C*2/*c*	*C*2/*c*	*C*2/*c*	*C*2/*c*	*C*2/*c*	*C*2/*c*	*C*2/*c*
T / K	100(2)	100(2)	100(2)	100(2)	100(2)	100(2)	100(2)
a / pm	1058.31(6)	1066.83(4)	1061.70(1)	1061.55(3)	1056.85(2)	1055.23(2)	1057.50(3)
b / pm	645.98(3)	655.43(3)	651.23(1)	649.97(2)	646.40(1)	643.81(1)	646.03(2)
c / pm	689.43(4)	694.41(3)	690.52(3)	689.59(2)	686.64(1)	685.31(1)	687.02(2)
β / °	92.048(5)	92.263(3)	92.258(3)	92.235(2)	92.174(2)	92.093(1)	92.101(3)
V / (10^6 pm^3)	471.03(4)	485.18(4)	477.03(1)	475.44(2)	468.74(1)	465.27(1)	469.04(2)
Z	4	4	4	4	4	4	4
$\rho_{calc.}$ / (g cm^{-3})	3.21	4.05	4.15	4.21	4.30	4.37	4.30
μ(Mo-K$_\alpha$) / mm^{-1}	12.5	13.8	14.9	15.8	17.0	18.1	17.0
Crystal size / μm^3	112 × 118 × 183	16 × 125 × 193	87 × 59 × 4	15 × 183 × 218	14 × 20 ×27	152 × 167 × 202	46 × 58 × 91
$2\theta_{max}$ / °	66.6	66.4	67.1	67.0	76.5	66.9	67.1
Index range	$-15 \le h \le 15$<br>$-9 \le k \le 9$<br>$-10 \le l \le 10$	$-16 \le h \le 16$<br>$-9 \le k \le 9$<br>$-10 \le l \le 10$	$-15 \le h \le 15$<br>$-9 \le k \le 9$<br>$-10 \le l \le 10$	$-15 \le h \le 15$<br>$-9 \le k \le 9$<br>$-10 \le l \le 10$	$-18 \le h \le 18$<br>$-11 \le k \le 11$<br>$-11 \le l \le 11$	$-16 \le h \le 16$<br>$-9 \le k \le 9$<br>$-10 \le l \le 10$	$-16 \le h \le 16$<br>$-9 \le k \le 9$<br>$-10 \le l \le 10$
Measured reflections	6244	7296	7945	7877	10758	7854	5493
Unique reflections	862	879	869	873	1246	864	893
R_{int},	0.017	0.018	0.017	0.020	0.056	0.017	0.034
R_σ	0.009	0.011	0.010	0.012	0.013	0.011	0.024
Extinction coefficient	–	$6.1(9) \cdot 10^{-3}$	$5.7(4) \cdot 10^{-3}$	$16(1) \cdot 10^{-3}$	–	$34(3) \cdot 10^{-3}$	–
Parameters	48	49	49	49	48	49	48
$R_1[F_o > 4\sigma(F_o)]$	0.0406	0.0243	0.0160	0.0259	0.0223	0.0394	0.0370
wR_2(all F_o^2)	0.1034	0.0734	0.0414	0.0765	0.0604	0.1204	0.0997
GooF	1.083	1.137	1.136	1.200	1.062	1.263	1.207
Residual electron density / (e 10^{-6} pm^{-3})	2.26 to –1.54	1.30 to –2.12	0.84 to –1.32	1.29 to –2.06	1.7 to –1.9	2.26 to –3.71	2.16 to –2.71

Table S3. Crystallographic data for $Na_2[Ho(BO_3)(OH)_2]$ at 310 K.

$Na_2RE[(BO_3)(OH)_2]$	*RE*=Ho
Crystal system	monoclinic
Space group	*Cmcm* (no. 63)
T / K	310(1)
a / pm<br>*b* / pm<br>*c* / pm<br>β / °<br>*V* / (10^6 pm^3)	1064.61(9)<br>645.74(8)<br>690.76(8)<br>90<br>474.88(9)
Z	4
$\rho_{calc.}$ / (g cm^{-3})	4.25
μ(Mo-K_α) / mm^{-1}	16.8
Crystal size / μm^3	139 × 88 ×78
$2\theta_{max}$ / °	71.3
Index range	$-17 \leq h \leq 17$<br>$-9 \leq k \leq 10$<br>$-11 \leq l \leq 11$
Reflections: measured, unique	8583, 594
R_{int}, R_σ	0.053, 0.016
Extinction coefficient	0.0109(5)
Parameters	34
$R_1[F_o > 4\sigma(F_o)]$	0.015
wR_2(all F_o^2)	0.035
GooF	1.317
Residual electron density / (e 10–6 pm–3)	1.89 to –1.87

Table S4. Monoclinic angles (space group *C*2/*c*) of $Na_2[RE(BO_3)(OH)_2]$ (*RE* = Y, Gd–Er) at room temperature (296 K).

RE	Y	Gd	Tb	Dy	Ho	Er	$Dy_{0.5}Er_{0.5}$
β / °	90.128(5)	90.143(2)	90.125(8)	90.513(3)	90.451 (2)	90.443(2)	90.202(4)

Table S5. Space group (SG), lattice parameters (/pm), β-angle (/°) and unit cell volumes (/10^6 pm^3) of Na_2**Ho**[$(BO_3)(OH)_2$] at different temperatures.

T (K)	SG	*a*	*b*	*c*	β	*V*
320	*Cmcm*	1064.42(10)	646.24(8)	691.12(8)	90	475.40(9)
310	*Cmcm*	1064.61(10)	645.74(8)	690.76(8)	90	474.88(9)
300	*C*2/*c*	1063.70(2)	645.82(1)	690.56(1)	90.445(2)	474.37(1)
290	*C*2/*c*	1063.52(2)	645.93(1)	690.43(1)	90.770(2)	474.25(1)
280	*C*2/*c*	1063.00(2)	645.74(1)	690.19(1)	90.979(2)	473.76(3)
270	*C*2/*c*	1062.70(2)	645.74(1)	689.93(1)	91.138(2)	473.36(2)
250	*C*2/*c*	1061.87(2)	645.81(1)	689.52(2)	91.371(2)	472.71(3)
200	*C*2/*c*	1059.66(2)	646.04(1)	688.68(1)	91.745(2)	471.24(1)
150	*C*2/*c*	1058.93(3)	646.19(2)	687.43(2)	92.031(2)	470.09(2)
100	*C*2/*c*	1056.85(2)	646.4(1)	686.6(1)	92.174(2)	468.74(1)

Table S6. Coordinates and coefficients U_{ij} (/ pm^2) of the tensor of the anisotropic displacement factor and equivalent or isotropic displacement factor for the atoms in β-Na_2[**Ho**$(BO_3)(OH)_2$] at 310 K (space group ***Cmcm***). U_{eq} is defined as one third of the trace of the orthogonalized U_{ij} tensor. All atoms occupy general Wyckoff positions.

Atom	*x*	*y*	*z*	U_{11}	U_{22}	U_{33}	U_{12}	U_{13}	U_{23}	U_{eq}, U_{iso}
Ho	0	0.12889(2)	¼	95.0(9)	62.0(10)	72.2(9)	0	0	0	76.4(7)
Na	0.1642(1)	½	0	172(5)	361(7)	165(5)	0	0	–79(5)	233(2)
O1	0.2125(3)	0.1600(5)	¼	102(8)	242(9)	221(10)	1(7)	0	0	6.7(6)
O2	0	0.1903(4)	0.5820(3)	401(12)	107(8)	81(8)	0	0	–16(6)	188(4)
O3	0	0.4839(5)	¼	152(10)	93(10)	117(10)	0	0	0	197(4)
B	0	0.6926(7)	¼	96(13)	52(14)	55(12)	0	0	0	68(5)
H	0.259(10)	0.287(12)	¼							900(200)

Table S7. Coordinates and coefficients U_{ij} (/ pm^2) of the tensor of the anisotropic displacement factor and equivalent or isotropic displacement factor for the atoms in α-Na_2[**Y**$(BO_3)(OH)_2$] at 100 K (space group ***C2/c***). U_{eq} is defined as one third of the trace of the orthogonalized U_{ij} tensor. All atoms occupy general Wyckoff positions.

Atom	*x*	*y*	*z*	U_{11}	U_{22}	U_{33}	U_{12}	U_{13}	U_{23}	U_{eq}, U_{iso}
Y	0	0.1291.0(5)	¼	99(2)	68(2)	124(2)	0	–8.0(14)	0	97.3(16)
Na	0.1645(11)	0.4811(2)	0.0125(2)	136(5)	155(6)	155(6)	3(4)	–5(4)	–18(4)	149(3)
O1	0.2126(2)	0.1616(4)	0.2383(4)	118(9)	113(8)	178(10)	–7(7)	–10(8)	–2(8)	137(4)
O2	0.0154(2)	0.1891(3)	0.5828(3)	198(11)	107(9)	121(9)	10(8)	–8(7)	–6(7)	142(4)
O3	0	0.4839(5)	¼	126(11)	75(11)	144(12)	0	–7(9)	0	115(5)
B	0	0.6947(7)	¼	98(17)	110(20)	152(18)	0	–23(14)	0	120(7)
H	0.249(5)	0.270(6)	0.281(8)							220(120)

Table S8. Coordinates and coefficients U_{ij} (/ pm^2) of the tensor of the anisotropic displacement factor and equivalent or isotropic displacement factor for the atoms in α-Na_2[**Gd**(BO_3)($OH)_2$] at 100 K (space group ***C*2/*c***). U_{eq} is defined as one third of the trace of the orthogonalized U_{ij} tensor. All atoms occupy general Wyckoff positions.

Atom	*x*	*y*	*z*	U_{11}	U_{22}	U_{33}	U_{12}	U_{13}	U_{23}	U_{eq}, U_{iso}
Gd	0	0.13014(3)	¼	62.7(17)	36.7(17)	66.7(17)	0	1.3(10)	0	554(13)
Na	0.1647(2)	0.4817(3)	0.0137(2)	93(7)	118(8)	93(7)	–2(6)	6(5)	–25(6)	101(3)
O1	0.2144(3)	0.1661(5)	0.2363(5)	72(12)	98(10)	120(13)	25(11)	3(10)	–28(13)	96(5)
O2	0.0191(3)	0.1913(4)	0.5839(4)	153(13)	40(12)	84(11)	–6(10)	29(9)	–1(8)	92(5)
O3	0	0.4866(6)	¼	98(16)	15(14)	111(16)	0	10(12)	0	74(6)
B	0	0.6932(10)	¼	40(20)	90(30)	70(20)	0	–30(2)	0	67(9)
H	0.272(11)	0.256(16)	0.28(2)							800(400)

Table S9. Coordinates and coefficients U_{ij} (/ pm^2) of the tensor of the anisotropic displacement factor and equivalent or isotropic displacement factor for the atoms in α-Na_2[**Tb**(BO_3)($OH)_2$] at 100 K (space group ***C*2/*c***). U_{eq} is defined as one third of the trace of the orthogonalized U_{ij} tensor. All atoms occupy general Wyckoff positions.

Atom	*x*	*y*	*z*	U_{11}	U_{22}	U_{33}	U_{12}	U_{13}	U_{23}	U_{eq}, U_{iso}
Tb	0	0.13003(2)	¼	50.7(11)	39.8(10	44.6(10)	0	5.4(5)	0	45.0(8)
Na	0.1647(1)	0.4816(2)	0.0137(1)	88(4)	119(4)	77(4)	–1(3)	10(3)	–15(3)	94.3(19)
O1	0.2142(2)	0.1651(3)	0.2366(3)	76(8)	106(6)	102(8)	–9(6)	7(6)	0(7)	94(3)
O2	0.0187(2)	0.1910(3)	0.5839(2)	158(8)	67(7)	66(7)	1(6)	21(6)	–2(6)	96(3)
O3	0	0.4866(4)	¼	85(9)	54(9)	64(10)	0	18(7)	0	67(4)
B	0	0.6954(5)	¼	57(13)	33(14)	76(14)	0	–13(10)	0	56(6)
H	0.269(5)	0.264(7)	0.26(1)							530(160)

Table S10. Coordinates and coefficients U_{ij} (/ pm^2) of the tensor of the anisotropic displacement factor and equivalent or isotropic displacement factor for the atoms in α-Na_2[**Dy**(BO_3)($OH)_2$] at 100 K (space group ***C*2/*c***). U_{eq} is defined as one third of the trace of the orthogonalized U_{ij} tensor. All atoms occupy general Wyckoff positions.

Atom	*x*	*y*	*z*	U_{11}	U_{22}	U_{33}	U_{12}	U_{13}	U_{23}	U_{eq}, U_{iso}
Dy	0	0.12978(3)	¼	81(2)	69.5(19)	74.0(18)	0	0.3(10)	0	74.9(15)
Na	0.1652(2)	0.4806(3)	0.0138(2)	121(7)	161(8)	99(6)	–2(6)	2(5)	–8(6)	127(3)
O1	0.2140(3)	0.1637(6)	0.2381(6)	84(13)	139(11)	135(14)	–5(11)	–1(1)	–11(13)	120(5)
O2	0.0174(3)	0.1906(5)	0.5832(4)	204(14)	113(13)	86(12)	–9(11)	21(10)	1(9)	134(5)
O3	0	0.4853(6)	¼	141(17)	69(15)	81(14)	0	17(12)	0	97(7)
B	0	0.6928(11)	¼	60(20)	120(30)	100(20)	0	2(17)	0	94(10)
H	0.295(15)	0.22(5)	0.23(6)							400(200)

Table S11. Coordinates and coefficients U_{ij} (/ pm^2) of the tensor of the anisotropic displacement factor and equivalent or isotropic displacement factor for the atoms in α-Na_2[**Ho**(BO_3)($OH)_2$] at 100 K (space group ***C*2/*c***). U_{eq} is defined as one third of the trace of the orthogonalized U_{ij} tensor. All atoms occupy general Wyckoff positions.

Atom	*x*	*y*	*z*	U_{11}	U_{22}	U_{33}	U_{12}	U_{13}	U_{23}	U_{eq}, U_{iso}
Ho	0	0.12955(2)	¼	52.9(8)	31.7(9)	40.1(8)	0	4.6(5)	0	41.5(6)
Na	0.1649(1)	0.4810(2)	0.0130(2)	93(4)	116(5)	81(5)	–3(3)	8(3)	–19(4)	97(2)
O1	0.2130(2)	0.1628(4)	0.2374(4)	69(7)	84(7)	101(9)	0(6)	6(6)	2(7)	85(3)
O2	0.0177(2)	0.1890(3)	0.5830(3)	150(8)	52(8)	57(7)	–5(6)	12(6)	1(6)	86(3)
O3	0	0.4856(4)	¼	92(10)	33(10)	66(10)	0	11(7)	0	63(4)
B	0	0.6970(6)	¼	81(13)	43(15)	59(14)	0	6(10)	0	61(6)
H	0.262(8)	0.270(11)	0.285(13)							360(170)

Table S12. Coordinates and coefficients U_{ij} (/ pm^2) of the tensor of the anisotropic displacement factor and equivalent or isotropic displacement factor for the atoms in α-Na_2[**Er**(BO_3)($OH)_2$] at 100 K (space group ***C*2/*c***). U_{eq} is defined as one third of the trace of the orthogonalized U_{ij} tensor. All atoms occupy general Wyckoff positions.

Atom	*x*	*y*	*z*	U_{11}	U_{22}	U_{33}	U_{12}	U_{13}	U_{23}	U_{eq}, U_{iso}
Er	0	0.12908(6)	¼	77(3)	56(3)	59(3)	0	7.8(17)	0	64(3)
Na	0.1649(3)	0.4804(5)	0.0134(4)	124(13)	123(13)	85(12)	4(10)	13(10)	–2(1)	111(6)
O1	0.2119(6)	0.160(1)	0.237(1)	80(20)	120(20)	130(30)	10(20)	–1(19)	2(2)	109(10)
O2	0.0168(6)	0.1883(9)	0.5818(8)	160(20)	110(2)	90(20)	–4(19)	46(18)	8(18)	119(9)
O3	0	0.488(2)	¼	110(30)	70(30)	60(30)	0	30(20)	0	80(12)
B	0	0.696(2)	¼	20(30)	90(50)	50(40)	0	–10(30)	0	55(17)
H	0.241(12)	0.290(8)	0.24(2)							0(200)

Table S13. Coordinates and coefficients U_{ij} (/ pm^2) of the tensor of the anisotropic displacement factor and equivalent or isotropic displacement factor for the atoms in α-Na_2[**$Dy_{0.5}Er_{0.5}$**(BO_3)($OH)_2$] at 100 K (space group ***C*2/*c***). U_{eq} is defined as one third of the trace of the orthogonalized U_{ij} tensor. All atoms occupy general Wyckoff positions.

Atom	*x*	*y*	*z*	U_{11}	U_{22}	U_{33}	U_{12}	U_{13}	U_{23}	U_{eq}, U_{iso}
Dy	0	0.12921(5)	¼	69(2)	26(2)	51(2)	0	5.9(12)	0	48.5(18)
Er	0	0.12921(5)	¼	69(2)	26(2)	51(2)	0	5.9(12)	0	48.5(18)
Na	0.1646(2)	0.4808(4)	0.0127(3)	125(10)	102(12)	90(10)	-8(8)	11(7)	–18(9)	106(5)
O1	0.2131(4)	0.1629(8)	0.2378(8)	82(18)	93(18)	100(20)	3(15)	1(15)	6(19)	91(8)
O2	0.0173(4)	0.1896(7)	0.5823(6)	171(19)	60(20)	73(19)	–5(15)	17(14)	–7(16)	101(7)
O3	0	0.4845(9)	¼	100(2)	30(20)	70(20)	0	0(17)	0	66(10)
B	0	0.699(2)	¼	120(40)	70(40)	100(40)	0	0(3)	0	96(16)
H	0.277(8)	0.254(17)	0.257(17)							200(200)

Table S14. Comparison of the coordinates and coefficients U_{ij} (/ pm^2) of the tensor of the anisotropic displacement factor and equivalent or isotropic displacement factor for the atoms in α-Na_2[**Ho**(BO_3)$(OH)_2$] at 100 K,150 K, 200 K, 250 K, 270 K, 280 K, 290 K, 300 K. All atoms occupy general Wyckoff positions.

Atom	*x*	*y*	*z*	U_{11}	U_{22}	U_{33}	U_{12}	U_{13}	U_{23}
Ho	0	0.12955(2)	¼	52.9(8)	31.7(9)	40.1(8)	0	4.6(5)	0
		0.1293.7(3)		51.7(14)	37.6(15)	51.1(16)		9.1(8)	
		0.12919(2)		62.2(12)	48.0(12)	60.1(12)		2.8(6)	
		0.12898(3)		82.7(12)	61.2(13)	80.7(13)	0	1.3(7)	0
		0.12889(3)		86.7(12)	59.4(12)	87.3(13)		–0.5(6)	
		0.12888(3)		82.5(11)	48.1(12)	77.3(12)		1.1(6)	
		0.12881(3)		94.6(11)	59.4(12)	85.9(12)		–02.8(6)	
		0.12885(2)		99.4(10)	56.2(11)	86.5(11)		–3.8(6)	
Na	0.1649(1)	0.4810(2)	0.0130(2)	93(4)	116(5)	81(5)	–3(3)	8(3)	–19(4)
	0.1646(2)	0.4822(3)	0.0122(2)	98(6)	153(8)	95(7)	–23(5)	10(5)	–23(5)
	0.1643(1)	0.4841(3)	0.0108(2)	121(6)	215(8)	113(6)	–08(5)	–02(4)	–37(5)
	0.1645(2)	0.4861(3)	0.0087(2)	150(7)	277(9)	156(7)	–08(6)	–10(5)	–50(6)
	0.1644(2)	0.4880(3)	0.0072(2)	160(7)	297(9)	176(7)	–59(6)	–09(5)	–59(6)
	0.1643(15)	0.4894(3)	0.0065(2)	156(7)	299(9)	169(7)	–0.0(6)	–4(5)	–62(6)
	0.1643(16)	0.4916(3)	0.0053(3)	177(7)	323(10)	181(7)	–16(6)	–2(6)	–78(7)
	0.1641(2)	0.4956(3)	0.0025(2)	180(6)	346(10)	183(7)	–13(6)	–08(5)	–78(6)
O1	0.2130(2)	0.1628(4)	0.2374(4)	69(7)	84(7)	101(9)	0(6)	6(6)	2(7)
	0.2127(3)	0.1613(6)	0.2393(5)	65(11)	117(10)	115(13)	7(10)	10(9)	3(11)
	0.2127(3)	0.1604(5)	0.2408(4)	83(10)	154(9)	139(11)	1(9)	4(8)	–1(9)
	0.2126(3)	0.1600(6)	0.2429(5)	100(10)	186(11)	172(13)	–3(10)	–8(9)	7(11)
	0.2124(3)	0.1595(5)	0.2436(5)	97(10)	197(11)	197(13)	1(9)	–3(9)	4(10)
	0.2125(3)	0.1591(5)	0.2449(5)	94(10)	188(11)	195(13)	–2(9)	–0(9)	–1(10)
	0.2122(3)	0.1594(6)	0.2456(5)	131(11)	215(12)	189(13)	4(10)	–7(10)	–1(11)
	0.2124(3)	0.1596(5)	0.2475(5)	114(10)	212(11)	233(14)	6(9)	1(9)	7(11)
O2	0.0177(2)	0.1890(3)	0.5830(3)	150(8)	52(8)	57(7)	–5(6)	12(6)	1(6)
	0.0156(3)	0.1889(4)	0.5824(4)	211(13)	75(12)	45(10)	–25(9)	31(9)	-20(8)
	0.0135(3)	0.1891(4)	0.5823(3)	261(13)	95(10)	70(9)	–11(8)	37(8)	–5(7)
	0.0097(3)	0.1891(4)	0.5826(4)	237(15)	94(11)	71(10)	–7(10)	15(9)	–23(8)
	0.0079(3)	0.1894(4)	0.5821(3)	380(16)	87(11)	85(10)	0(1)	15(9)	–19(8)
	0.0068(3)	0.1901(4)	0.5820(4)	364(15)	77(11)	85(10)	–15(10)	9(9)	–9(8)
	0.0052(3)	0.1898(4)	0.5809(4)	392(16)	94(11)	86(10)	–4(10)	19(10)	–17(8)
	0.0028(3)	0.1894(4)	0.5812(4)	393(15)	109(11)	89(10)	1(10)	–1(10)	–31(8)
O3	0	0.4856(4)	¼	92(10)	33(10)	66(10)	0	11(7)	0
		0.4841(6)		107(14)	34(15)	94(15)		18(11)	
		0.4851(5)		98(12)	50(13)	89(12)		06(9)	
		0.4844(6)		132(13)	67(14)	106(13)		16(10)	
		0.4836(5)		133(13)	71(13)	127(13)		3(10)	
		0.4837(5)		145(13)	44(13)	125(13)		7(10)	
		0.4836(5)		155(14)	81(14)	113(13)		–6(10)	
		0.4849(5)		152(13)	81(14)	146(14)		–3(10)	
B	0	0.6970(6)	¼	81(13)	43(15)	59(14)	0	6(10)	0
		0.694(1)		76(19)	80(2)	80(2)		3(15)	
		0.6946(7)		69(15)	43(18)	80(16)		2(12)	
		0.6958(9)		105(18)	100(2)	74(17)		10(13)	
		0.6954(9)		102(17)	130(2)	88(18)		–3(13)	
		0.6949(8)		116(17)	80(2)	73(17)		–9(13)	
		0.6946(8)		120(17)	80(2)	90(18)		–3(13)	
		0.6956(7)		121(16)	62(18)	46(15)		13(12)	

H	0.262(8)	0.270(11)	0.285(13)
	0.246(14)	0.236(13)	0.27(2)
	0.262(6)	0.251(8)	0.274(9)
	0.256(12)	0.249(12)	0.268(16)
	0.253(14)	0.244(14)	0.270(19)
	0.257(13)	0.240(13)	0.261(19)
	0.259(17)	0.27(2)	0.27(3)
	0.253(11)	0.216(17)	0.225(15)

Table S15. Comparison of the coordinates and coefficients U_{ij} (/ pm^2) of the tensor of the anisotropic displacement factor and equivalent or isotropic displacement factor for the atoms in Na_2[**Ho**$(BO_3)(OH)_2$] at 310 K and 320 K. All atoms occupy general Wyckoff positions.

Atom	x	y	z	U_{11}	U_{22}	U_{33}	U_{12}	U_{13}	U_{23}	U_{eq}, U_{iso}
Ho	0	0.12890(2)	¼	95.0(9)	62.0(10)	72.2(9)	0	0	0	76.4(7)
		0.12890(4)		130.2(19)	84.9(18)	104.8(17)	0	0	0	106.6(14)
Na	0.1642(1)	½	0	172(5)	361(7)	165(5)	0	0	–79(5)	233(2)
	0.1642(2)			230(1)	382(12)	196(9)	0	0	–80(8)	269(5)
O1	0.2125(3)	0.1600(5)	¼	102(8)	242(9)	221(10)	1(7)	0	0	6.7(6)
	0.2123(4)	0.1610(8)		129(16)	259(16)	279(19)	0(14)	0	0	222(7)
O2	0	0.1903(4)	0.5820(3)	401(12)	107(8)	81(8)	0	0	–16(6)	188(4)
		0.1906(5)	0.5821(5)	450(2)	133(15)	117(13)	0	0	–12(11)	234(8)
O3	0	0.4839(5)	¼	152(10)	93(10)	117(10)	0	0	0	197(4)
		0.4851(8)		200(2)	123(18)	136(17)	0	0	0	152(8)
B	0	0.6926(7)	¼	96(13)	52(14)	55(12)	0	0	0	68(5)
		0.6934(10)		150(3)	70(3)	90(2)	0	0	0	104(10)
H	0.259(10)	0.287(12)	¼							900(200)
	0.255(14)	0.290(16)								400(200)
